# Regulatory switch at the cytoplasmic interface controls TRPV channel gating

**DOI:** 10.7554/eLife.47746

**Published:** 2019-05-09

**Authors:** Lejla Zubcevic, William F Borschel, Allen L Hsu, Mario J Borgnia, Seok-Yong Lee

**Affiliations:** 1 Department of BiochemistryDuke University School of MedicineDurhamUnited States; 2 Genome Integrity and Structural Biology Laboratory, Department of Health and Human ServicesNational Institute of Environmental Health Sciences, National Institutes of HealthDurhamUnited States; National Institute of Neurological Disorders and Stroke, National Institutes of HealthUnited States; The University of Texas at AustinUnited States

**Keywords:** TRP channel, ion channel, ligand dependent gating, sensitization, cryo-electronmicroscopy, electrophysiology, Human

## Abstract

Temperature-sensitive transient receptor potential vanilloid (thermoTRPV) channels are activated by ligands and heat, and are involved in various physiological processes. ThermoTRPV channels possess a large cytoplasmic ring consisting of N-terminal ankyrin repeat domains (ARD) and C-terminal domains (CTD). The cytoplasmic inter-protomer interface is unique and consists of a CTD coiled around a β-sheet which makes contacts with the neighboring ARD. Despite much existing evidence that the cytoplasmic ring is important for thermoTRPV function, the mechanism by which this unique structure is involved in thermoTRPV gating has not been clear. Here, we present cryo-EM and electrophysiological studies which demonstrate that TRPV3 gating involves large rearrangements at the cytoplasmic inter-protomer interface and that this motion triggers coupling between cytoplasmic and transmembrane domains, priming the channel for opening. Furthermore, our studies unveil the role of this interface in the distinct biophysical and physiological properties of individual thermoTRPV subtypes.

## Introduction

**Figure 1.**
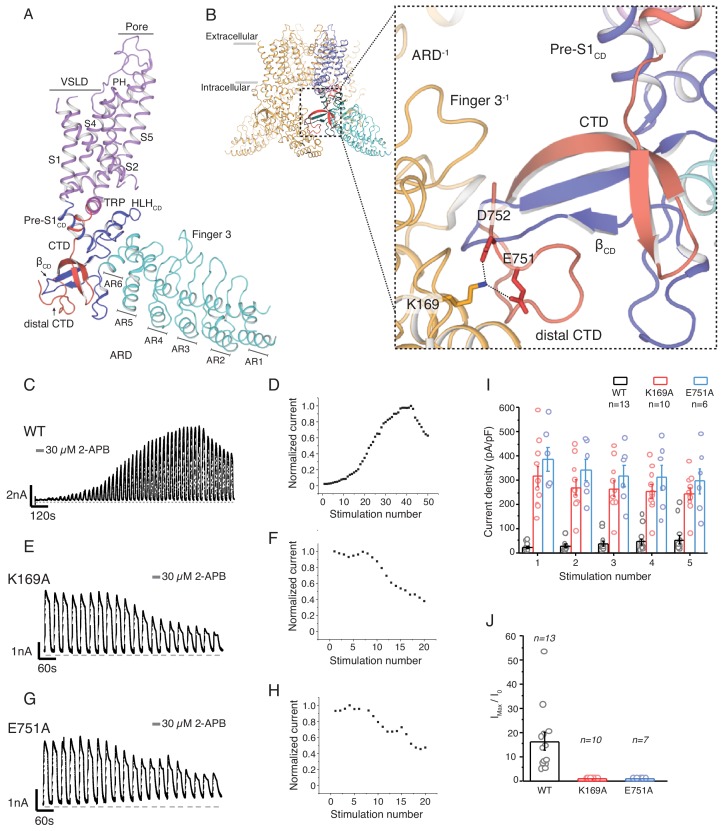
The role of the cytoplasmic inter-protomer interface in hTRPV3 gating. (**A**) Architecture of the hTRPV3 protomer. Ankyrin repeat domain (ARD) is colored in cyan, the coupling domain (CD) and its individual elements (HLH_CD_, β_CD_, Pre-S1_CD_) are colored in blue, transmembrane helices S1-S6 are colored in violet, the TRP domain is shown in magenta and the C-terminal domain (CTD) is colored in red. (**B**) A close-up view of the inter-protomer interface in hTRPV3. Residue K169 from the ARD and residues E751 and D752 from the CTD are shown in stick representation. Representative whole-cell current traces recorded at +60 mV from WT (**C–D**), K169A (**E–F**), and E751A (**G–H**) evoked by repeating applications 30 μM 2-APB for 15 s followed by 15 s of washout and corresponding time-course of use-dependent changes in the relative current amplitude. (**I**) Average current density from the first five 2-APB stimulations (WT: n = 13 biologically independent experiments; K169A: n = 10 biologically independent experiments; E751A: n = 6 biologically independent experiments). (**J**) Initial sensitization was characterized by the ratio of the response to 2-APB during the first (I_0_) and maximum current (I_max_) response (I_max_/I_0_) calculated as the mean from each biologically independent experiment. (WT: n = 13 biologically independent experiments; K169A: n = 10 biologically independent experiments; E751A: n = 7 biologically independent experiments). 10.7554/eLife.47746.004Figure 1—source data 1.This spreadsheet contains data points for the time course of 2-APB use-dependence plots in Figure 1D, 1F and 1H; current density data used to generate bar plots in Figure 1I; initial sensitization data used to generate bar plots in Figure 1J.

The Transient Receptor Potential Vanilloid (TRPV) channel subfamily is a subset of the large TRP channel superfamily, and consist of subtypes TRPV1-TRPV6 (Gunthorpe et al., 2002). TRPV1-4 are intrinsically temperature sensitive (thermoTRPV) (Caterina et al., 1997; Caterina et al., 1999; Güler et al., 2002; Smith et al., 2002) and have been found to play important roles in numerous physiological processes including thermosensation (Moqrich et al., 2005; Marics et al., 2014; Patapoutian, 2005; Gavva et al., 2008), nociception (Caterina et al., 2000; Gopinath et al., 2005; Bang et al., 2010; Reilly and Kym, 2011; Huang and Chung, 2013), and osmosensation (Zanou et al., 2015). Recent studies have made great strides in elucidating the molecular mechanisms of ligand-dependent gating and activation of thermoTRPV channels (Hu et al., 2009; Cao et al., 2013; Zhang et al., 2016; Zubcevic et al., 2018a; Zubcevic et al., 2018b; Singh et al., 2018; Zhang et al., 2019). However, despite this wealth of structural information, the role of their cytoplasmic domains, which make up the majority of the structure, remains unclear. The importance of these domains in channel gating has long been acknowledged with a number of studies finding that mutations in the cytoplasmic regions can profoundly affect the function of thermoTRPV channels (Lishko et al., 2007; Phelps et al., 2010; Shi et al., 2013; Landouré et al., 2010; Salazar et al., 2008; Yao et al., 2011; Brauchi et al., 2006). Cryo-electron microscopy (cryo-EM) and X-ray crystallography studies (Cao et al., 2013; Zubcevic et al., 2018a; Zubcevic et al., 2018b; Singh et al., 2018; Liao et al., 2013; Zubcevic et al., 2016; Huynh et al., 2016; Deng et al., 2018) have revealed that the cytoplasmic domains of thermoTRPVs are composed of the ankyrin repeat domain (ARD), containing six ankyrin repeats, a coupling domain (CD) that is made up of a β-sheet (β_CD_), a helix-loop-helix motif (HLH_CD_) and the pre-S1(pre-S1_CD_) helix, and a C-terminal domain (CTD) which extends from the conserved amphipathic helix, termed the TRP domain, into the cytosol where it forms a hair-pin structure and doubles back to the β_CD_ to which it contributes a beta strand (Figure 1A). Interestingly, a recent study of the human TRPV3 (hTRPV3) channel (Zubcevic et al., 2018b) resolved the structure of the CTD beyond the β_CD_ and showed that this distal region of the CTD (distal CTD) coils around β_CD_ and emerges at the front side of the channel. This coil forms extensive interactions with the ARD of the neighboring protomer and therefore contributes substantially to the cytoplasmic inter-protomer interface. All thermoTRPV channels possess cytoplasmic inter-protomer contacts which are formed by the ARD of one and the CD of the neighboring protomer and the distal CTD region is conserved in these channels. However, a distal CTD coil similar to the one observed in TRPV3 was only seen in TRPV2 (Zubcevic et al., 2018a) since this region was poorly resolved in the structures of TRPV1 and TRPV4 (Cao et al., 2013; Liao et al., 2013; Deng et al., 2018).

**Figure 1—figure supplement 1.**
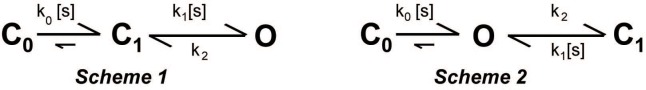
State model and gating schemes of TRPV3 channels. Simple state model of TRPV3 gating proposed by Liu et al. (2011). The transition rate from C_1_ → C_0_ (scheme 1) and O→C_0_ (scheme 2) is slow enough that this transition is irreversible. Both k_0_ and k_1_ transition rates are stimulant [s] dependent (heat or chemical agonist). Following removal of the stimulus, channels return to a closed intermediate state (C_1_) in scheme 1 or a new resting state (C_1_) in scheme 2. During consecutive stimulations, the occupancy of C_1_ increases, leading to greater sensitivity to the stimulus in a use-dependent manner.

In order to dissect the role of this unique cytoplasmic interface in thermoTRPV channel gating, we have conducted structural and functional studies using human TRPV3 as a model system. TRPV3 exhibits use-dependent increase in current amplitudes (termed sensitization) upon repeated applications of either ligand or heat. This phenomenon arises from hysteresis (irreversible change) of TRPV3 gating and can be adequately described by a simple gating scheme with one open and two closed states (Liu et al., 2011) (Figure 1—figure supplement 1). Following stimulation, the return rate to the initial unliganded closed state (C_0_) of the channel is slow enough to be irreversible. Instead, the channels transition to either an intermediate (sensitized) closed state (C_1_ in Scheme 1, Figure 1—figure supplement 1) or a new sensitized resting state after opening (C_1_ in Scheme 2, Figure 1—figure supplement 1). Repeated stimulation leads to an increase in C_1_ occupancy reflected in progressive increase in current in response to stimuli. Therefore, the C_1_ state represents a closed state with a lower energy requirement for opening. Sensitization properties of thermoTRPVs are strongly subtype dependent: while TRPV3 (Liu et al., 2011) and TRPV2 (Liu and Qin, 2016) both sensitize upon repeated stimulation with heat or agonists, analogous sensitization upon stimulation with capsaicin or heat has not been observed in TRPV1, suggesting that TRPV1 channels do not undergo an irreversible conformational change following activation by a stimuli, and might populate a closed ‘sensitized’ C_1_ state from the outset (Liu and Qin, 2016).

Our structural and functional studies show that the cytoplasmic assembly plays a critical role in activation of thermoTRPV channels through large structural rearrangements at the CTD-ARD interface that involve secondary structure transitions. The data also provides an explanation for the phenotypic differences amongst the thermoTRPV channels and sheds light on the role of the cytoplasmic inter-protomer interface in thermoTRPV gating.

## Results

### Disrupting the interactions between CTD and ARD results in sensitized channels

Our previously determined structures of the hTRPV3 channel revealed that the distal CTD coils around β_CD_ and establishes a number of interactions with the ARD of the neighboring protomer (Zubcevic et al., 2018b). Notably, the acidic residues E751 and D752 in the distal CTD form a salt-bridge with the K169 residue on the neighboring ARD (Figure 1B). A previous study reported that mutation of K169A altered sensitivity to ligands and proposed allosteric modulators of TRPV3 channel function; however, the underlying mechanism was elusive as the study was conducted prior to structural elucidation of the full-length TRPV channels (Phelps et al., 2010). With the knowledge that K169 forms a part of the CTD-ARD interface, we set out to investigate its role in channel gating. Wild-type hTRPV3 channels show a steady use-dependent increase in the current response upon successive applications of 30 μM 2-Aminoethoxydiphenyl borate (2-APB) for ~30–40 cycles of stimulation before reaching saturation (Figure 1C–D, Figure 1—source data 1). By contrast, the K169A mutant appears to be fully sensitized, as the first few applications of ligand typically elicit the maximal current response which does not increase upon further stimulation, suggesting the channel is sensitized (Figure 1E–F, Figure 1—source data 1). In order to determine if this sensitized phenotype is the result of disruption of the salt-bridge interaction between ARD and CTD, we introduced a E751A mutation in the CTD of the hTRPV3 channel. Indeed, similar to K169A, neutralizing the acidic E751 residue produced a sensitized phenotype, characterized by large saturating currents upon first application of ligand (Figure 1G–H, Figure 1—source data 1). Together these data suggest that the salt-bridge formed by K169 and E751 at the CTD-ARD interface plays a critical role in hysteresis and that breaking these interactions changes the occupancy of the C_0_ and C_1_ states before stimulation, resulting in sensitized hTRPV3 channels (Figure 1I–J, Figure 1—source data 1).

### Structure of the K169A mutant reveals large conformational changes in the distal CTD

**Figure 2.**
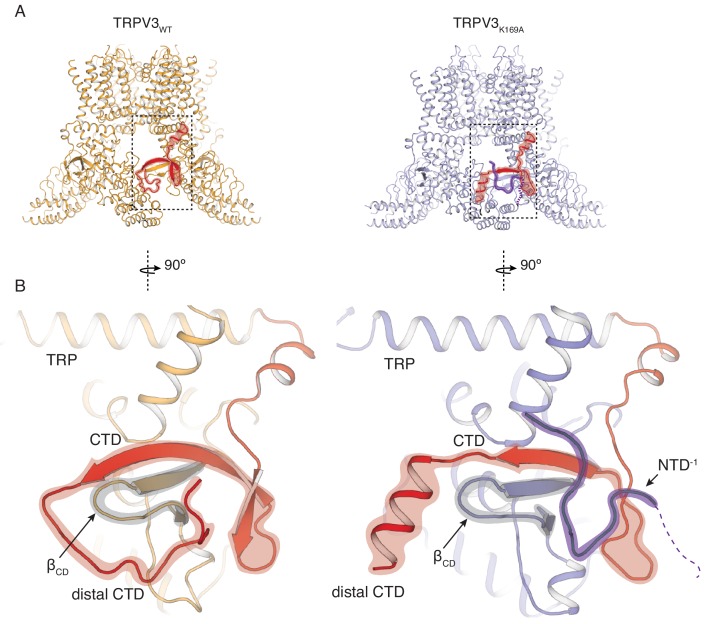
Rearrangements of the cytoplasmic domains in the TRPV3_K169A_ structure. (**A**) The cytoplasmic inter-protomer interface in TRPV3_WT_ (left panel) and TRPV3_K169A_ (right panel). The CTD and the putative N-terminal region are highlighted in red and purple, respectively. (**B**) Close-up view of the rearrangements in the cytoplasmic domains. In the TRPV3_WT_, the distal CTD (highlighted in red) coils around the β_CD_ (highlighted in grey) (left panel). In the TRPV3_K169A_ structure, the distal CTD undergoes a coil-to-helix transition (highlighted in red). An additional polypeptide density (highlighted in purple) is observed near the front of the β_CD_ (highlighted in grey) and the proximal CTD, in the vicinity of the space occupied by the distal CTD coil in TRPV3_WT_ and was assigned as a putative N-terminal domain from the neighboring protomer (NTD^−1^).

In order to elucidate the conformational changes that underlie the transition to the sensitized C_1_ state, we introduced the K169A mutation into the hTRPV3 carrying the previously reported functionally silent T96A mutation (Zubcevic et al., 2018b) (TRPV3_K169A_ in future references). We expressed and purified the TRPV3_K169A_ channel and solved its structure by single particle 3D cryo-electron microscopy (cryo-EM) (Table 1, Figure 2—figure supplements 1 and 2). Remarkably, the cytoplasmic assembly of TRPV3_K169A_ undergoes large structural rearrangements while the overall conformation of the transmembrane domains (TM) of the TRPV3_K169A_ channel closely resembles that of the previously reported hTRPV3 in its closed, apo form (PDB ID 6MHO, TRPV3_WT_) (Zubcevic et al., 2018b) (Figure 2A). The most drastic change occurs at the CTD. In the TRPV3_WT_, the distal CTD coils around β_CD_ and establishes a large interface between the CD and the ARD of neighboring subunits. By contrast, the K169A mutation induces a substantial change in the secondary structure and the position of the distal CTD as well as the ARD-CTD interface. Specifically, the distal CTD undergoes a dramatic coil-to-helix transition and this newly formed helical distal CTD is positioned behind the β_CD_ in the cytoplasmic vestibule of the channel (Figure 2B, Figure 2—figure supplement 2). Furthermore, the cryo-EM map of the TRPV3_K169A_ contains a protein density that abuts the proximal CTD and the CD and occupies a similar space to that vacated by the distal CTD upon coil-to-helix transition. It is unlikely that this density forms a part of the CTD as the cryo-EM map shows no connectivity to the CTD helix. Instead, the density appears to be connected to the N-terminal of the neighboring protomer to which connectivity is visible at low contours of the map (Figure 2B, Figure 2—figure supplement 2). Since this density is not sufficiently resolved to allow for unambiguous model building, we built this putative N-terminal region as a polyalanine chain.

**Figure 2—figure supplement 1.**
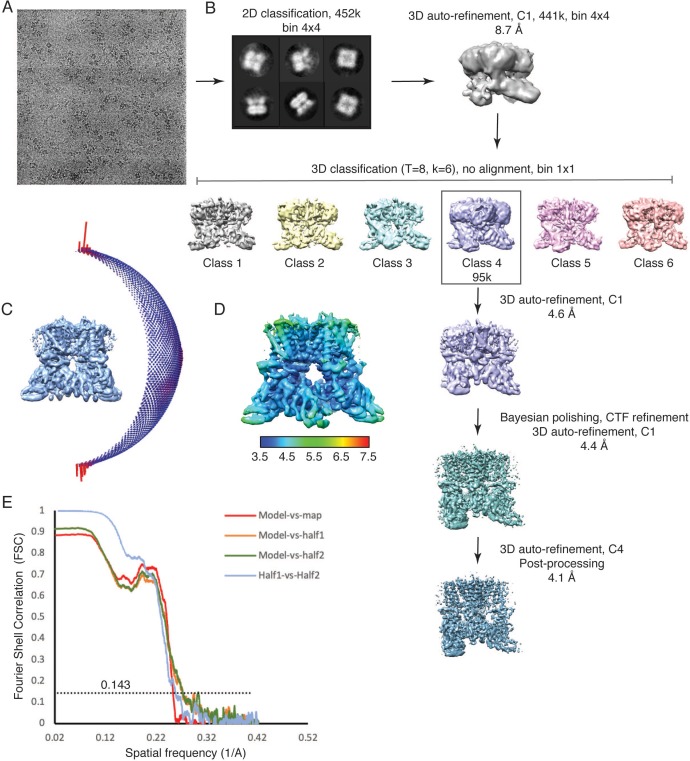
Cryo-EM data collection and processing, TRPV3_K169A_. (**A**) A representative micrograph from the TRPV3_K169A_ data collection. (**B**) 3D reconstruction workflow. (**C**) Euler distribution plot. Red regions show the best represented views. (**D**) Local resolution estimate, calculated in Relion. (**E**) FSC curves calculated between the half maps (blue), atomic model and the full map (red) and between the model and each half-map (orange and green).

**Figure 2—figure supplement 2.**
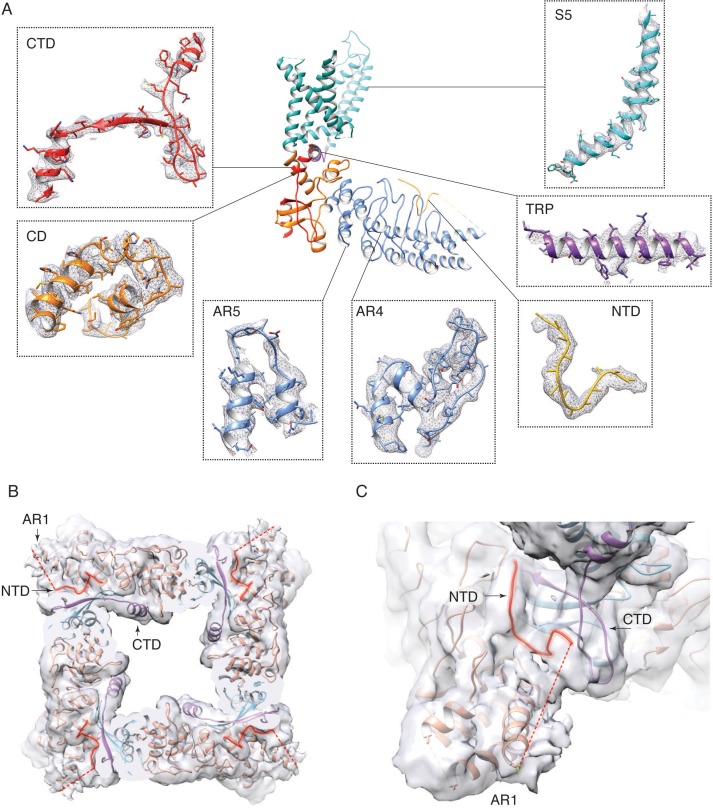
Electron density in the TRPV3_K169A_ cryo-EM map. (**A**) Representative electron densities in the TRPV3_K169A_ cryo-EM map. Densities are contoured at level 0.015 and radius 2. (**B–C**) Connectivity between the ARD and the putative N-terminal region. The unsharpened TRPV3_K169A_ map is contoured at level 0.006.

**Table 1.** Cryo-EM data collection, refinement and validation statistics

	TRPV3_K169A_ (EMD-20192) (PDB 6OT2)	TRPV3_K169A 2-APB_ (EMD-20194) (PDB 6OT5)
Data collection and processing		
Magnification	130,000x	75,000x
Voltage (kV)	300	300
Electron exposure (e–/Å^2^)	40	42
Defocus range (μm)	1–2.5	1.25–3
Pixel size (Å)	1.06	1.08
Symmetry imposed	C4	C4
Initial particle images (no.)	452,388	1,174,521
Final particle images (no.)	95,184	79,006
Map resolution (Å)	4.1	3.6
FSC threshold	0.143	0.143
Refinement		
Initial model used (PDB code)	6MHO	6MHO
Model resolution (Å)	4.1	3.6
FSC threshold	0.143	0.143
Map sharpening *B* factor (Å^2^)	−120	−100
Model composition		
Non-hydrogen atoms	17,332	17,800
Protein residues	2500	2492
Ligands	0	4 (2-APB)
*B* factors (Å^2^)		
Protein	87.43	40.51
Ligand	n/a	35.66
R.m.s. deviations		
Bond lengths (Å)	0.008	0.008
Bond angles (°)	0.868	0.833
MolProbity score	1.64	1.24
Clashscore	5	5
Poor rotamers (%)	0	0
Ramachandran plot		
Favored (%)	92.70	97.01
Allowed (%)	7.30	2.99
Disallowed (%)	0	0

### Conformational changes of the distal CTD lead to rearrangements in the cytoplasmic inter-subunit interface

**Figure 3.**
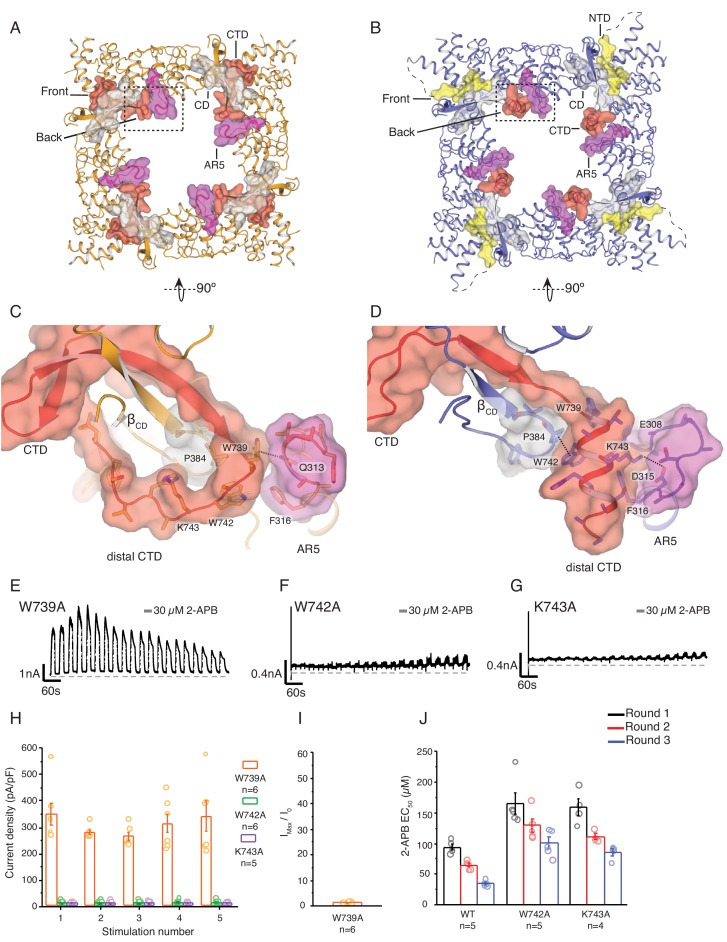
State-dependent changes at the cytoplasmic inter-protomer interface. (**A–B**) Top view of the cytoplasmic inter-protomer interactions in TRPV3_WT _(**A**) and TRPV3_K169A_ (**B**). In TRPV3_WT_ the CTD (red) coils around the β_CD_ (grey). The distal CTD interacts with the ARD at the front of the interface and with the loop of ankyrin repeat 5 (AR5, magenta) at the back. In TRPV3_K169A_, the interface is changed due to the coil-to-helix transition in the distal CTD, which no longer participates in the interactions at the front of the interface and forms tighter interactions with AR5. The front of the interface is now occupied by the putative NTD (yellow). (**C**) A close-up view from the cytoplasmic cavity of the interactions between the distal CTD (red surface representation) and AR5 (magenta surface representation) in TRPV3_WT_. Residue W739 forms a cation-π interaction with the amino group of Q313 (dashed line). (**D**) The coil-to-helix transition changes the conformation of the AR5 loop. In TRPV3_K169A_, the W739-Q313 interaction is broken. Residues K742 and W743, which in TRPV3_WT_ are not within interaction distances with the rest of the protein, form interactions with the backbone of E308 in AR5 and P384 in β_CD,_ respectively (dashed lines). Representative whole-cell current traces recorded at +60 mV from W739A (**E**), W742A (**F**), and K743A (**G**) evoked by repeating applications 30 μM 2-APB for 15 s followed by 15 s of washout. (**H**) Average current density for the first five 2-APB stimulations (W739A: n = 6 biologically independent experiments; W742A: n = 6 biologically independent experiments; K743A: n = 5 biologically independent experiments). (**I**) Ratio of first (I_0_) and maximum current (I_max_) 2-APB stimulation (I_max_/I_0_) as in (**G**), calculated as the mean from each biologically independent experiment (W739A: n = 6 biologically independent experiments). (**J**) Mean 2-APB EC_50_ from three consecutive dose-response rounds fit with the Hill equation (WT: n = 5 biologically independent experiments; W742A: n = 5 biologically independent experiments; K743A: n = 4 biologically independent experiments). See Figure 3—figure supplement 2 for representative current traces and dose–response relationship fit with the Hill equation. 10.7554/eLife.47746.013Figure 3—source data 1.This spreadsheet contains current density data used to generate bar plots in Figure 3H; initial sensitization data used to generate bar plots in Figure 3I; calculated dose-response values used to generate bar plots in Figure 3J.

The coil-to-helix transition in the distal CTD and the rearrangement of the putative N-terminal region in the TRPV3_K169A_ are accompanied by an apparent anti-clockwise rotation of the ARD when viewed from the extracellular space (Figure 3—figure supplement 1). This rotation does not reflect a strict rigid body movement of the ARD as the tetrameric assembly of TRPV3_K169A_ cytoplasmic domains cannot be superposed well with that of TRPV3_WT_ through mere rotation (Cα R.M.S.D. 2.3 Å) (Figure 3—figure supplement 1). Nevertheless, individual ARDs from the two structures superpose well (Cα R.M.S.D. 0.9 Å) (Figure 3—figure supplement 1), revealing that the ARDs of each TRPV3_K169A_ protomer swivel in a manner which lifts the N-terminal part of the ARD towards the membrane while the C-terminal part of the ARD along with the CTD is lowered further into the cytosol (Figure 3—figure supplement 1). Furthermore, the coil-to-helix transition also results in extensive changes in the interface between ARD and CTD (Figure 3—figure supplement 1), causing a conformational change of the loop of ankyrin repeat 5 (AR5) (Figure 3A and Figure 3—figure supplement 1). The combined effect of these rearrangements increases the coupling between the CTD and the ARD, as well as between the ARD, the CD and the TRP domain (Figure 3—figure supplement 1).

**Figure 3—figure supplement 1.**
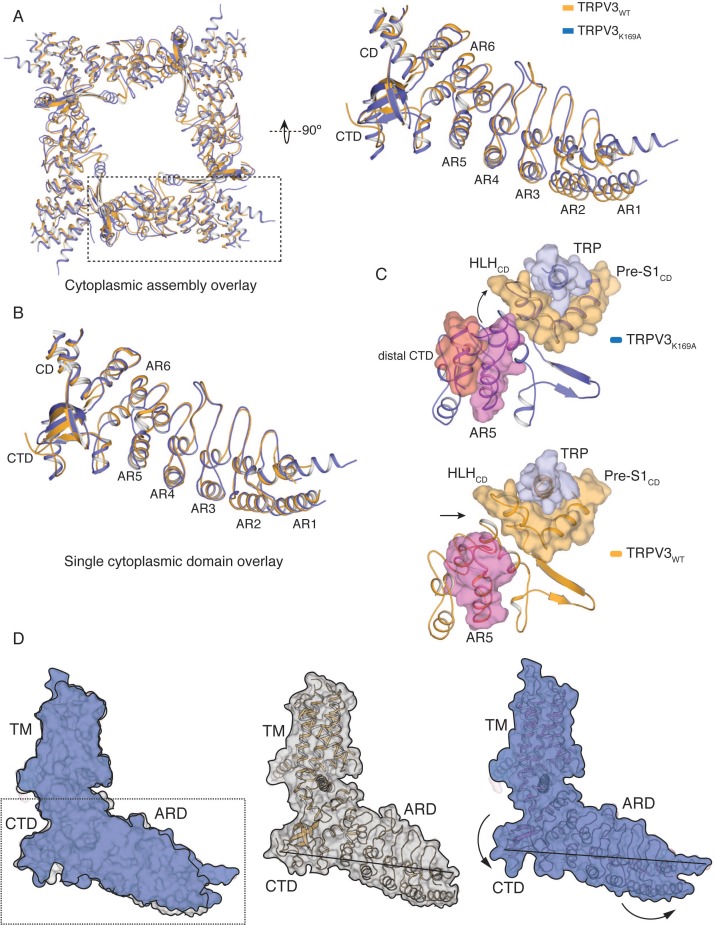
Conformational changes in the cytoplasmic domains in TRPV3_K169A_. (**A**) Overlay of the tetrameric cytoplasmic assemblies from TRPV3_WT_ (orange) and TRPV3_K169A_ (blue). Close-up view single ARD shows that the tetrameric assemblies cannot be superposed through a rigid body rotation. (**B**) Individual ARDs, not attached to the tetrameric assembly, from TRPV3_WT_ (orange) and TRPV3_K169A_ (blue) can be superposed. (**C**) In the TRPV3_WT_ (orange, bottom) structure there is no coupling between the AR5 (magenta) and the HLH_CD_ (orange). However, in TRPV3_K169A_ (blue, top) upon coil-to-helix transition in the distal CTD (red), the CTD-AR5 (magenta) interaction forces a change in conformation in the loop of AR5, bringing it within interaction distance of HLH_CD_ (orange). (**D**) Surface representation of the overlay between single protomers of TRPV3_WT_ (orange cartoon, gray surface) and TRPV3_K169A_ (blue cartoon, blue surface). The overlay shows that the TM domains overlay well, while the cytoplasmic domains exhibit substantial differences. The cytoplasmic assembly of TRPV3_K169A_ (right panel) swivels, so that the N-terminal part is lifted up toward the membrane while the C-terminal part is lowered into the cytosol. The line drawn between the tip of the β_CD_ and residue A110 in AR1 indicates the relative movement within the cytoplasmic assembly.

**Figure 3—figure supplement 2.**
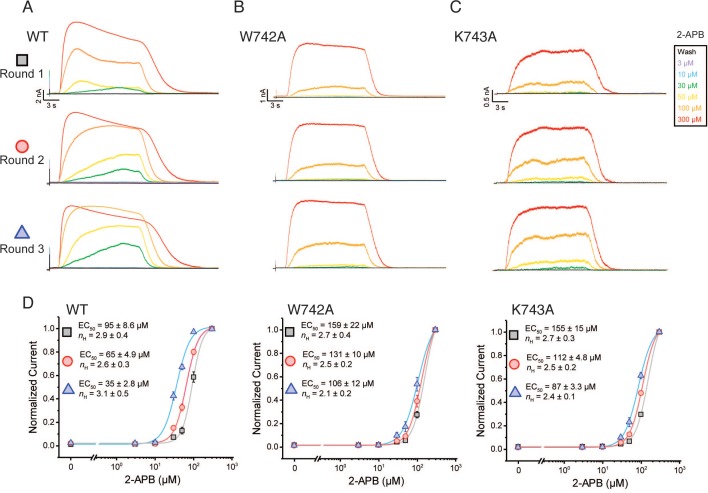
The TRPV3 W742A and K743A CTD mutants alter hysteresis. Three consecutive rounds of 2-APB dose-responses (0 (*black*) 3(*purple*), 10 (*blue*), 30 (*green*), 50 (*yellow*), 100 (*orange*), 300 (*red*) μM) was applied for 15 s followed 15 s of wash for WT (**A**) W742A (**B**) and K743A (**C**) at +60 mV. (**D**) Averaged dose-response of WT, W742A, and K743A from the first (square), second (circle), and third (triangle) consecutive dose-response round. *Inset* is EC_50_ and hill coefficient (*n*_H_) fitted to the average normalized current at each 2-APB concentration per round (WT: n = 5 biologically independent experiments; W742A: n = 5 biologically independent experiments; K743AA: n = 4 biologically independent experiments). 10.7554/eLife.47746.012Figure 3—figure supplement 2—source data 1.This spreadsheet contains dose-response data used to calculate mean values plotted in Figure 3—figure supplement 2D.

In the TRPV3_WT_ structure, the distal CTD makes contacts with ankyrin repeat 2 (AR2) and the loop connecting AR3 and AR4, termed finger 3, at the front side of the interface (Figure 1B) and the loop of AR5 in the intracellular vestibule (the back side of the interface) (Figure 3A and C). By contrast, in the TRPV3_K169A_ the front side of the interface appears to be formed by the N-terminal region, which extends from the neighboring ARD and abuts the CD and the proximal CTD (Figures 2B and 3B). Notably, while the distal CTD of TRPV3_K169A_ does not contribute to the front side of the interface with minimal interaction with the CD of its own protomer, it forms extensive interactions with the loop of AR5 of the neighboring subunit at the back side of the interface (Figure 3B and D). In order to probe the role of the dynamic interactions between the ARD and CTD observed in TRPV3_K169A_, we introduced mutations in the distal CTD and examined their effects on channel function. We chose three sites W739, W742, and K743 for mutational studies. In TRPV3_WT_, W739 forms a cation-π interaction with the amino group of Q313 in the loop of AR5 (Figure 3C), and this interaction is broken in the TRPV3_K169A_ (Figure 3D). Similarly, both W742 and K743 undergo state-dependent changes in their interactions: when the distal CTD adopts a coil conformation W742 and K743 do not form substantial interactions with the rest of the channel (Figure 3C), but when the distal CTD is helical W742 forms a CH-π interaction with P384 in the CD and K743 is within interaction distance of E308 and D315 in the loop of AR5 (Figure 3D). The W739A mutation resulted in a phenotype similar to the sensitized K169A and E751A mutants (Figure 3E). By contrast, W742A (Figure 3F) and K743A (Figure 3G) both resulted in channels with markedly lower activity and decreased ability to sensitize upon repeated stimulation with 30 μM 2-APB (Figure 3H, Figure 3—source data 1). This reduced activity was not the result of decreased surface expression or non-functional channels, as application of 2-APB at high concentrations (300 μM) resulted in robust current responses (Figure 3—figure supplement 2). To further probe this low activity phenotype of W742A and K743A, we examined the effects of these mutations on hysteresis. As the wild-type TRPV3 undergoes hysteresis, its sensitivity to ligand increases, which can be monitored by the reduction of the EC_50_ value (Figure 3—figure supplement 2). The initial 2-APB EC_50_ values for both W742A and K743A were higher than that for the wild-type TRPV3 channel (Figure 3J; Figure 3—figure supplement 2, Figure 3—source data 1, Figure 3—figure supplement 2—source data 1), but they decreased following each consecutive dose-response round. However, because the 2-APB dose-response curves for W742A and K743A mutants do not reach saturation, the efficacy of 2-APB is likely to be overestimated by these EC_50_ calculations. These results indicate that W742A and K743A are initially less active due to impaired sensitization and might have an increased energy barrier for transitioning to the sensitized conformation. Combined with the results from E751A and K169A, these data show that mutations to different parts of the distal CTD have distinct effects on sensitization and activation: mutations that destabilize the coil conformation of the distal CTD result in a sensitized phenotype, while mutations that destabilize the helical CTD result in a decrease of hysteresis and sensitization. Taken together, our data suggest that the coil-to-helix transition in the distal CTD and the resulting state-dependent inter-protomer interaction networks are critical for channel gating and that the secondary structure transition in the distal CTD may serve as a switch that controls the entry into the sensitized C_1_ state.

### Application of 2-APB induces changes in both transmembrane and cytoplasmic assemblies

**Figure 4.**
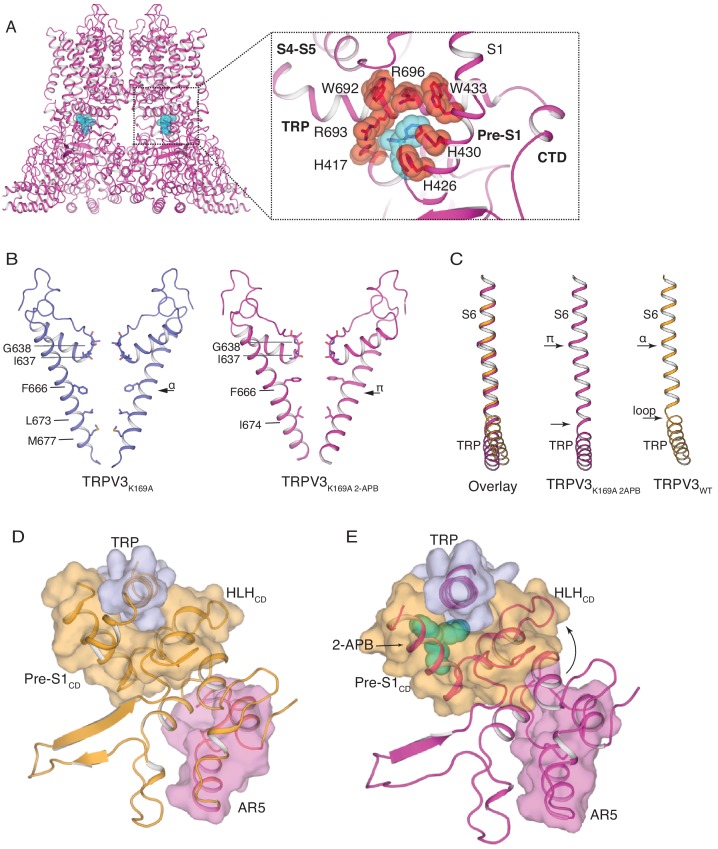
The structure of TRPV3_K169A 2-APB_ exhibits changes in both transmembrane and cytoplasmic domains. (**A**) One 2-APB molecule is bound to each protomer of the TRPV3_K169A 2-APB_ channel (magenta). 2-APB is found between the HLH_CD_, Pre-S1_CD_ and TRP domains in a binding site defined by residues H417 in the HLH_CD_, H426, H430, W433 in the Pre-S1_CD_ and W692, R693 and R696 in the TRP domain. All residues are shown in stick and red sphere representation. 2-APB is shown in stick and cyan sphere representation. (**B**) The S6 helix of TRPV3_K169A_ (blue) undergoes an α-to-π transition in the presence of 2-APB (magenta). (**C**) The α-to-π transition tightens the connection between S6 and the TRP domain. In the TRPV3_WT_ structure (orange), the TRP domain and the S6 are connected via a loop, but in the TRPV3_K169A 2-APB_ channel (magenta) the TRP domain and S6 form a continuous helical structure. In addition, the TRP domain exhibits a swivel in the TRPV3_K169A 2-APB_ structure. (**D–E**) The coil-to-helix transition in TRPV3_K169A 2-APB_ increases coupling between the cytoplasmic domains and the TRP domain. In the TRPV3_WT_ structure (orange) (**D**), the loop of AR5 (magenta surface) does not interact with the HLH_CD_ (orange surface). However, in TRPV3_K169A 2-APB_ (magenta) (**E**) the coil-to-helix transition in the distal CTD induces a conformational change in the loop of AR5 (magenta surface), coupling it to the HLH_CD_ (orange surface) and the TRP domain (light blue surface). 2-APB (cyan stick and surface representation) contributes to increased interactions between the TRP domain and Pre-S1_CD_.

In order to further investigate the role of the cytoplasmic assembly in activation of the TRPV3 channel, we determined the cryo-EM structure of the TRPV3_K169A_ channel in the presence of 2-APB (TRPV3_K169A 2-APB_) to 3.6 Å resolution (Figure 4—figure supplements 1 and 2). Inspection of this structure revealed that TRPV3 undergoes conformational changes in both the transmembrane (TM) and cytoplasmic domains in the presence of ligand. The cryo-EM map of TRPV3_K169A 2-APB_ contains non-protein densities between the TRP domain and the Pre-S1_CD_ in all four protomers. Because a previous high-throughput mutagenesis study (Hu et al., 2009) identified this site as critical for 2-APB binding, we assigned the non-protein densities as 2-APB (Figure 4A, Figure 4—figure supplement 2). The 2-APB molecule is nestled amongst residues W692, R693 and R696 in the TRP domain and H417, H426, H430 and W433 in the Pre-S1_CD_.

**Figure 4—figure supplement 1.**
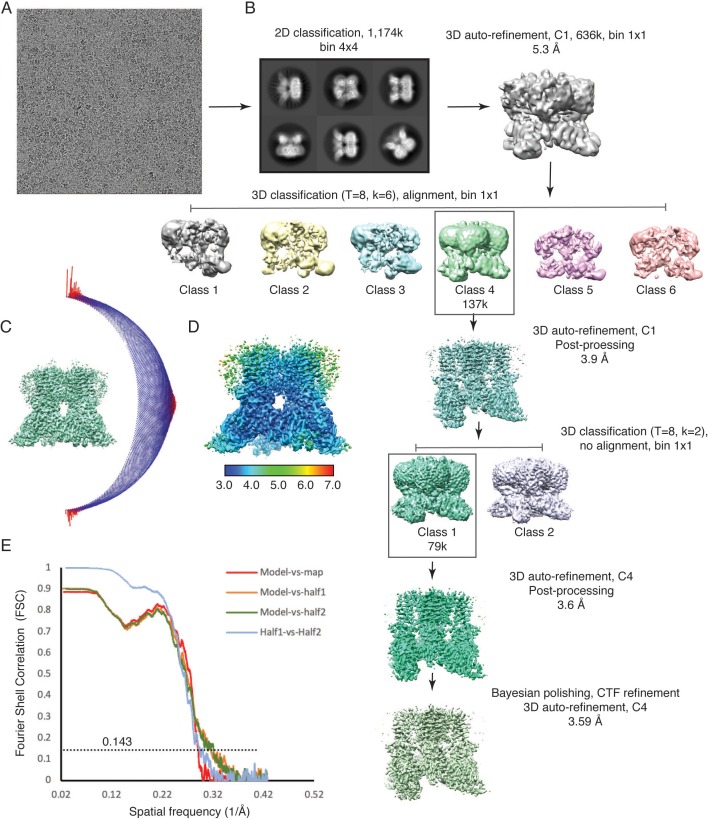
Cryo-EM data collection and processing, TRPV3_K169A 2-APB_. (**A**) A representative micrograph from TRPV3_K169A 2-APB_ data collection. (**B**) 3D reconstruction workflow. (**C**) Euler distribution plot. Red regions show the most represented views. (**D**) Local resolution estimate, calculated in Relion. (**E**) FSC curves calculated between the half maps (blue), atomic model and the full map (red) and between the model and each half-map (orange and green).

**Figure 4—figure supplement 2.**
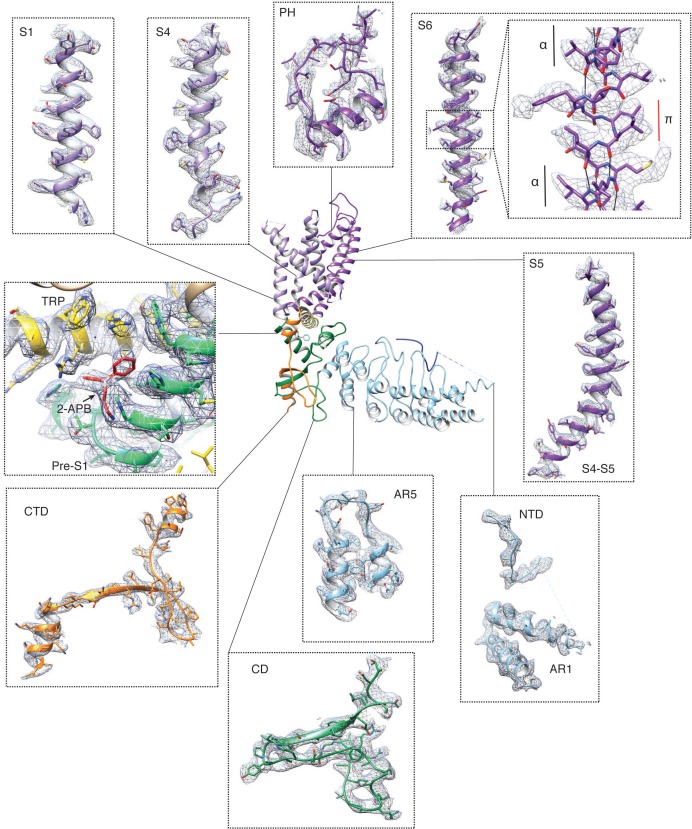
Electron density in the TRPV3_K169A 2-APB_ cryo-EM map. Representative electron densities in the TRPV3_K169A 2-APB_ cryo-EM map. Densities are contoured at level 0.03 and radius 2. The inset in the S6 panel shows a close-up of the density around the π-helical turn contoured at level 0.02 and radius 2. The black solid lines represent the H-bonds in the α-helix, which are broken in the π-helical turn.

**Figure 4—figure supplement 3.**
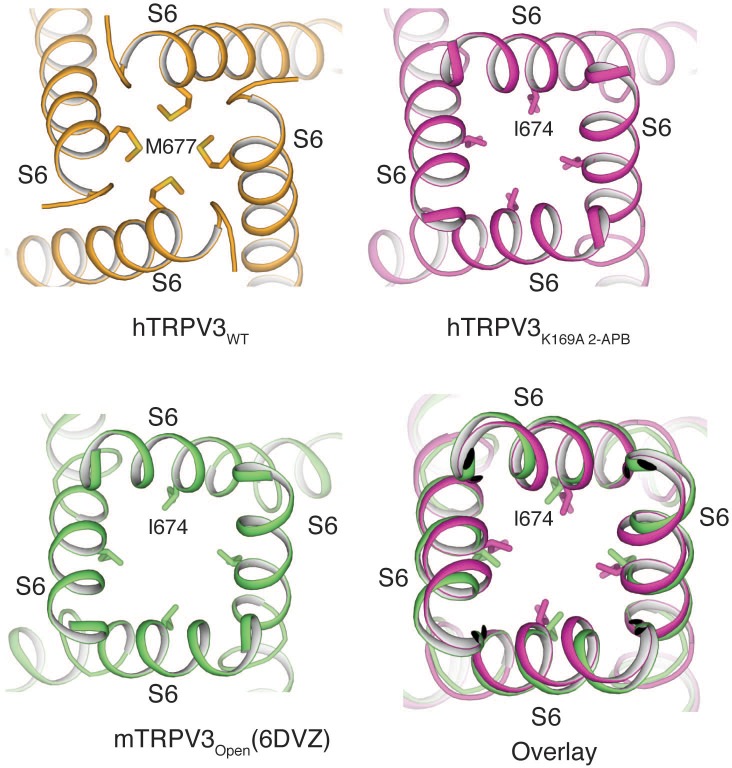
Comparison of the pore conformations of hTRPV3_WT_, hTRPV3_K169A 2-APB_ and mTRPV3_Open_. Bottom-up view of the hTRPV3_WT_ (PDB ID 6MHO, orange), hTRPV3_K169A 2-APB_ (magenta) and mTRPV3_Open_ (PDB ID 6DVZ, green) pores. The conformation of hTRPV3_K169A 2-APB_ resembles that of the mTRPV3_Open_ (Overlay).

**Figure 4—figure supplement 4.**
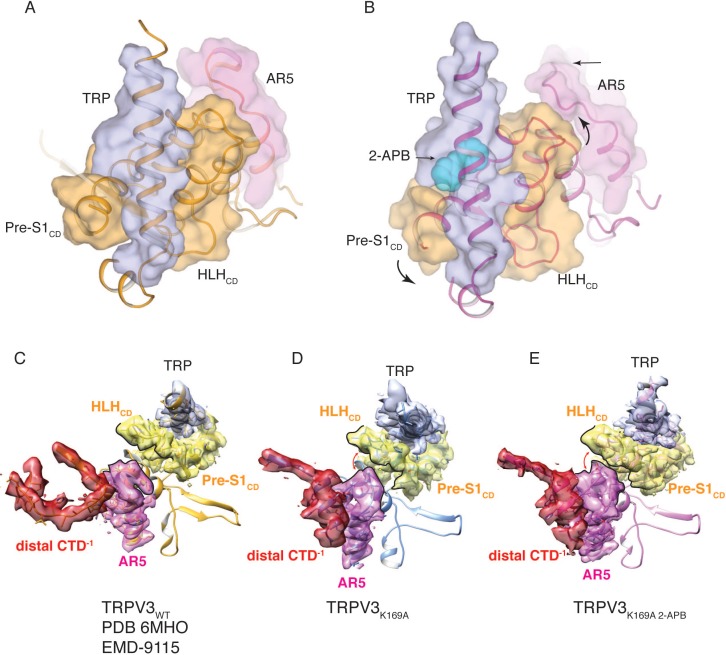
Coupling between the cytoplasmic and transmembrane domains. (**A**) Top view of the coupling between the TRP domain (light blue), the CD (orange, Pre-S1_CD_ and HLH_CD_) and the AR5 (magenta) in TRPV3_WT_. (**B**) In the TRPV3_K169A 2-APB_ structure, the coil-to-helix transition in the CTD forces the loop AR5 to change conformation (magenta) and interact with HLH_CD_ (orange). This induces a swivel in the CD (orange) and the TRP domain (light blue). 2-APB (cyan spheres) further increases the coupling between the CD and the TRP domain. (**C–E**) Electron density around the CTD, AR5, CD and TRP domain in TRPV3_WT_ (**C**), TRPV3_K169A_ (**D**) and TRPV3_K169A 2APB_ (**E**) viewed from inside of the cytoplasmic vestibule. The density is contoured at level 0.02 in TRPV3_WT_, 0.01 in TRPV3_K169A_ and 0.02 in TRPV3_K169A 2-APB_.

**Figure 4—figure supplement 5.**
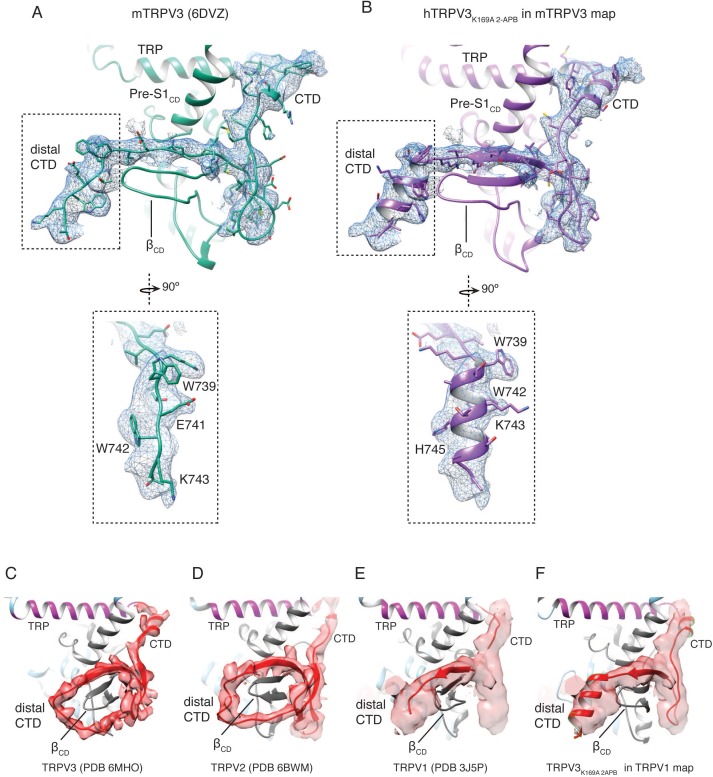
The CTD in thermoTRPV structures. (**A**) The cryo-EM map (EMD-8921) and atomic structure of the open mTRPV3 (PDB 6DVZ). Close-up view shows the fit of the CTD region into the electron density. (**B**) hTRPV3_K169A 2-APB_ fit into the cryo-EM map of the open mTRPV3 (EMD-8921). Close-up view shows that the electron density can more feasibly be built as a helix. (**C**) The cryo-EM map (EMD-9115) and atomic structure of the hTRPV3 apo, closed state (PDB ID 6MHO). (**D**) The X-ray crystallographic electron density and atomic structure of the apo closed rabbit TRPV2 (PDB ID 6BWM). (**E**) The cryo-EM map (EMD-5778) and atomic structure of the apo, closed rTRPV1 (PDB ID 3J5P). (**F**) hTRPV3_K169A 2-APB_ fit into the cryo-EM map of the open apo, closed rTRPV1 (EMD-5778).

**Figure 4—figure supplement 6.**
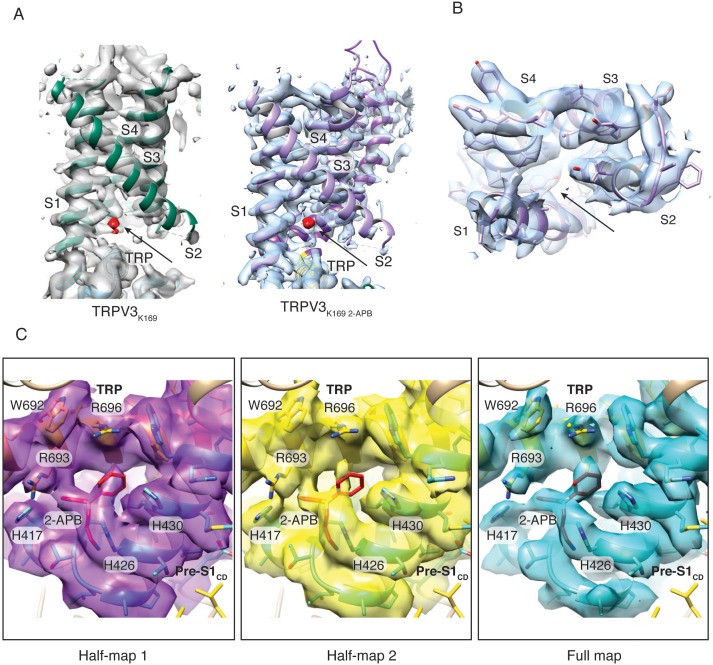
APB binding in TRPV3_K169A 2APB_. (**A**) Non-assigned densities (red) in the VSLD cavity of TRPV3_K169A_ (green, left) and TRPV3_K169A 2-APB_ (purple, right). Electron densities are contoured at levels 0.015 and 0.03, respectively. (**B**) No electron density is present at the proposed third 2-APB binding site at the extracellular side of the VSLD (arrow). Electron density is contoured at level 0.03. (**C**) Binding of 2-APB (red stick representation) between the TRP domain (yellow) and Pre-S1_CD_ (green) in TRPV3_K169A 2-APB_ half-map 1 (magenta, contoured at 0.0175), half-map 2 (yellow, contoured at 0.0175) and full map (blue, contoured at 0.03).

**Figure 4—figure supplement 7.**
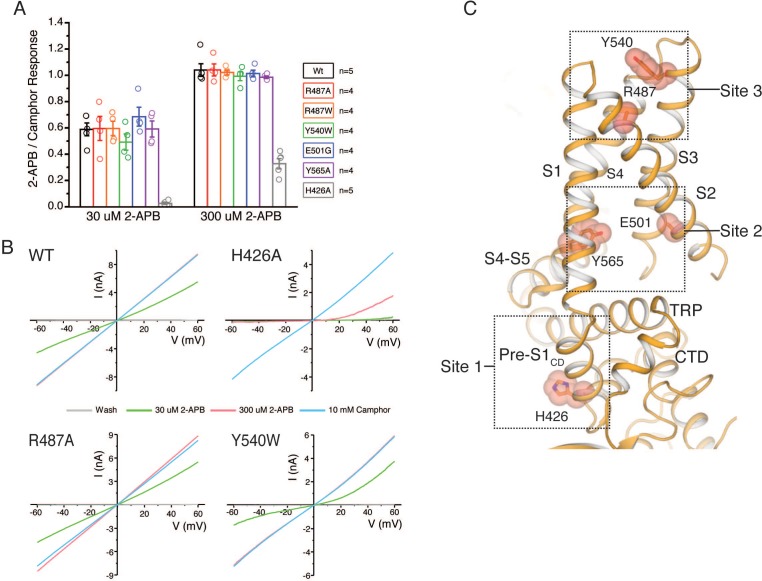
APB and camphor response of proposed 2-APB binding site mutants. (**A**) Graphical representation of the current response ratio of sub- (30 μM) and saturating 2-APB (300 μM) to camphor (10 mM) calculated as the mean from each biologically independent experiment. (WT: n = 5 biologically independent experiments; R487A: n = 4 biologically independent experiments; R487W: n = 4 biologically independent experiments; Y540W: n = 4 biologically independent experiments; E501G: n = 4 biologically independent experiments; Y540A: n = 4 biologically independent experiments; Y565A: n = 4 biologically independent experiments; H426A: n = 5 biologically independent experiments;). (**B**) Representative voltage ramp (−60 to +60 mV, 400 ms) traces to wash (*gray*), 30 (*green*) and 300 (*red*) μM 2-APB, and 10 mM camphor (*blue*). Only H426A possessed a lower 2-APB to camphor ratio compared to WT channels. (**C**) Protomer of mouse TRPV3 Y654A mutant bound to 2-APB (PDB ID 6DVZ) with three proposed binding sites highlighted (dotted boxes) (Singh et al., 2018). Mutated residues are shown in stick and sphere representation. 10.7554/eLife.47746.022Figure 4—figure supplement 7—source data 1.This spreadsheet contains data used to calculate the mean 2-APB to camphor ratio values plotted in Figure 4—figure supplement 7A.

The pore of TRPV3_K169A 2-APB_ adopts a putative open conformation similar to that of the recently reported mouse TRPV3 (mTRPV3, PDB ID 6DVZ)) open structure (Figure 4—figure supplement 3) and possesses a π-helical turn in the pore-lining S6 helix, which is not present in any structures of the TRPV3 channel that had not been exposed to ligand (Zubcevic et al., 2018b; Singh et al., 2018), including TRPV3_K169A_ (Figure 4B and Figure 4—figure supplement 2). Notably, a comparison of S6 helices from the closed TRPV3_WT_ and TRPV3_K169A 2-APB_ shows that the linker region between the S6 helix and the TRP domain also changes: while the two helices are connected via a loose loop in the TRPV3_WT_ structure, they form a single helical structure in TRPV3_K169A 2-APB_, suggestive of increased coupling between the TRP domain and S6 upon 2-APB binding (Figure 4C). In addition, the TRP domain of TRPV3_K169A 2-APB_ undergoes a swivel relative to the S6 helix (Figure 4C). A closer inspection revealed that the observed swivel in the TRP domain is the result of state-dependent changes in coupling between the transmembrane and the cytoplasmic domains. Namely, the swivel in the TRP domain can be traced back to changes in coupling between the loop of AR5, the HLH_CD_ and the TRP domain which are induced by the coil-to-helix transition in the distal CTD (Figure 4D–E). In TRPV3_WT_ the loop of AR5 does not interact with HLH_CD_ (Figure 4D and Figure 4—figure supplement 4). However, in TRPV3_K169A 2-APB_, where the coil-to-helix transition in the distal CTD has enforced a conformational change in the loop of AR5, AR5 interacts with the HLH_CD_. The loop of AR5 pushes on the HLH_CD_, causing a swivel in both the HLH_CD_ and Pre-S1_CD_, leading in turn to a swivel in the TRP domain (Figure 4E and Figure 4—figure supplement 4). Notably, 2-APB binding between the Pre-S1_CD_ and the TRP domain appears to further increase the coupling between the CD and the TRP domain, suggesting a mechanism for 2-APB-dependent activation of TRPV3 (Figure 4—figure supplement 4). In line with our observations, a previous study has shown that manipulation of the length of the loop of HLH_CD_ affects sensitization properties of TRPV3 likely by increasing the coupling between the cytoplasmic and transmembrane domains (Liu and Qin, 2017).

Our studies suggest that the distal CTD in TRPV3 plays a critical role in hysteresis, sensitization, and activation. In potential conflict with our findings, the previously reported open structural model of the mutant mouse TRPV3 (mTRPV3) in complex with 2-APB (PDB ID 6DVZ) contains a CTD in the loop conformation (Figure 4—figure supplement 5) (Singh et al., 2018). However, a closer inspection of the accompanying cryo-EM map (EMD-8921) revealed that this distal CTD density, assigned as a loop in the original study, could more feasibly be built as a helix (calculated correlation around mean equals 0.33 for the loop, and 0.49 for the helix, see Materials and methods, Figure 4—figure supplement 5). This further supports our finding that the coil-to-helix transition in the distal CTD is critical for channel activation.

Furthermore, the mTRPV3 study suggested three binding sites for 2-APB: (1) between the TRP domain and Pre-S1_CD_, (2) in the VSLD cavity and (3) at the extracellular interface between helices S1 and S3 of the VSLD (Singh et al., 2018). Even though we observe a non-protein density in the VSLD cavity of TRPV3_K169A 2-APB,_ we did not assign this to 2-APB because our reconstruction of TRPV3_K169A_ also contains a similarly shaped density in this position (Figure 4—figure supplement 6). Furthermore, we do not observe a discernible density in the third site proposed by the mTRPV3 study (Figure 4—figure supplement 6). Finally, electrophysiological measurements indicate that mutations in hTRPV3 at the proposed second and third sites do not affect the relative responses of 2-APB compared to camphor. By contrast, mutating residues in the first site (H426A) reduces the channels’ response to high concentrations of 2-APB without affecting the response to camphor (Figure 4—figure supplement 7, Figure 4—figure supplement 7—source data 1). This suggests that sites 2 and 3 are not involved in 2-APB-dependent activation of hTRPV3. These discrepancies in 2-APB binding between the human and mouse TRPV3 orthologs might be due to differential ligand affinities in different species. However, the role of site 2 and site 3 in 2-APB-dependent gating of mTRPV3 still remains to be electrophysiologically confirmed (Singh et al., 2018).

### The role of the distal CTD in activation of thermoTRPV

**Figure 5.**
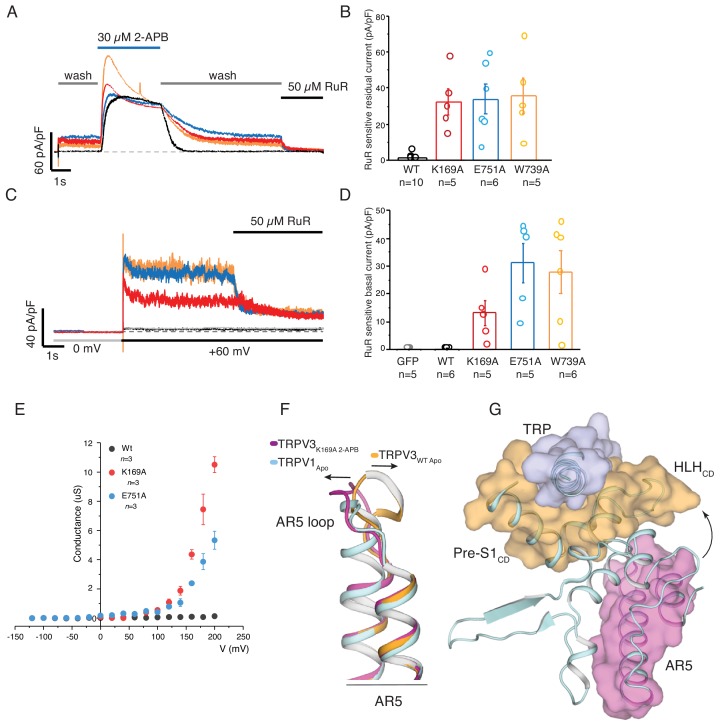
Parallels between TRPV3_K169A_ and wild-type TRPV1. (**A**) Representative whole-cell recording at +60 mV of WT (*black*), K169A (*red*), E751A (*blue*), W739A (*orange*) immediately following 2-APB sensitization protocol, with perfusion protocol of 5 s wash, followed by 15 s 30 μM 2-APB, prolonged 30 s wash and the residual current was blocked by 5 s application of 50 μM ruthenium red (RuR). The scale bars units are in current density (pA/pF). (**B**) Graphical representation of RuR sensitive residual current at the end of the recording in (**A**) (WT: n = 10 biologically independent experiments; K169A: n = 5 biologically independent experiments; E751A: n = 6 biologically independent experiments, W739A: n = 5 biologically independent experiments). (**C**) Basal current activity before application of ligand was determined by the blocking the current following a voltage step from 0 to +60 mV with 50 μM RuR. (**D**) Graphical representation of the mean blocked current density from the protocol in (**C**) (GFP control: n = 5 biologically independent experiments; WT: n = 6 biologically independent experiments; K169A: n = 5 biologically independent experiments; E751A: n = 5 biologically independent experiments, W739A: n = 6 biologically independent experiments). (**E**) Conductance voltage relation of WT, K169A, and E751A in the absence of ligand determined from peak tail current elicited from a −160 mV post step pulse following a voltage step between −120 to +200 mV (Δ 20 mV). The conductance of both mutants fail to saturate at +200 mV while WT channels are nonconductive at the tested voltages (WT: n = 3 biologically independent experiments; K169A: n = 3 biologically independent experiments; E751A: n = 3 biologically independent experiments). (**F**) Alignment of AR5 from TRPV3_WT_ (orange), TRPV3_K169A 2APB_ (magenta) and TRPV1 (light cyan). The AR5 loop of TRPV1 assumes a conformation similar to that of TRPV3_K169A 2APB_. (**G**) The AR5 loop and the HLH_CD_ are within interaction distance in TRPV1. 10.7554/eLife.47746.025Figure 5—source data 1.This spreadsheet contains the data used to calculate the ruthenium red sensitive current plots in Figure 5B and 5D; voltage step data used to calculate conductance plot in Figure 5E.

Following removal of 2-APB, the sensitized K169A, E751A, and W739A mutants retained residual activity after a prolonged washout of ligand (Figure 5A). This residual current, which might result from either an extremely slow 2-APB off-rate or constitutive activity at +60 mV, was sensitive to the pore blocker ruthenium red (RuR) (Figure 5B, Figure 5—source data 1). A voltage step from 0 to +60 mV prior to application of ligand elicited RuR sensitive currents (Figure 5C,D, Figure 5—source data 1) not seen in wild-type TRPV3 channels, suggesting that these mutants are inherently voltage gated in the absence of ligand. Notably, the G-V curve of the K169A and E751A mutants shows that voltage can directly activate the mutant channels (Figure 5E, Figure 5—source data 1), which is in stark contrast to the wild-type TRPV3 in which voltage is not sufficient for channel activation (Hu et al., 2009; Xu et al., 2002). Interestingly, the sensitized phenotype and voltage-dependent activation of the K169A and E751A TRPV3 bear resemblance to the behavior of the wild-type TRPV1 (Gunthorpe et al., 2000; Voets et al., 2004; Sánchez-Moreno et al., 2018), implying a mechanistic link in the activation of these two channels. A sequence comparison of thermoTRPV channels shows that the cytoplasmic inter-protomer interface is conserved and, remarkably, the distal CTD region in these channels exhibits high helical propensity (Figure 5—figure supplement 1). In order to examine the role of the distal CTD in the gating of thermoTRPVs, we inspected the cryo-EM map and model of the apo TRPV1 channel (Liao et al., 2013). Interestingly, we found striking similarities between the apo TRPV1 channel (PDB ID 3J5P) and the TRPV3_K169A-2APB_ structure. Firstly, the conformation of the loop of AR5 in apo TRPV1 is similar to that observed in TRPV3_K169A-2APB_ (Figure 5F). In addition, the cryo-EM density for the distal CTD, despite being poorly resolved, is compatible with a helical conformation of this region (Figure 4—figure supplement 5). Consequently, apo TRPV1 apparently exhibits tight coupling between the loop of AR5, the HLH_CD_ and the TRP domain (Figure 5G). Furthermore, the pore lining S6 helix of apo TRPV1 adopts a π-helical turn (Liao et al., 2013). Therefore, it appears that the cytoplasmic and transmembrane domains in the TRPV1 channel are tightly connected even in the absence of stimuli, possibly explaining the absence of a sensitizing phenotype in this channel as well as its ability to be activated by voltage. Intriguingly, the K155A mutation in TRPV1, analogous to K169A in TRPV3, reduces ligand-induced desensitization of TRPV1 (Lishko et al., 2007; Joseph et al., 2013) and replacement of the distal CTD in TRPV1 with that of TRPV3 produces channels that do not desensitize upon repeated application of heat (Joseph et al., 2013). By contrast, deletion of the TRPV1 CTD helix produces non-functional channels, indicating that the distal CTD and the cytoplasmic inter-protomer interface is critical for gating and activation of TRPV channels (Joseph et al., 2013). The non-sensitized closed structures (Zubcevic et al., 2018a; Zubcevic et al., 2018b) of the sensitizing TRPV2 and TRPV3 (Liu et al., 2011; Liu and Qin, 2016) channels, possess CTDs in a coil conformation and a lower degree of coupling between the cytoplasmic and transmembrane domains (Figure 4—figure supplement 5). Therefore, our structural and functional analyses support this novel hypothesis that the distal CTD acts as a conformational switch in thermoTRPVs, which is imperative for coupling between the cytoplasmic and transmembrane domains and consequently also for channel activation.

**Figure 5—figure supplement 1.**
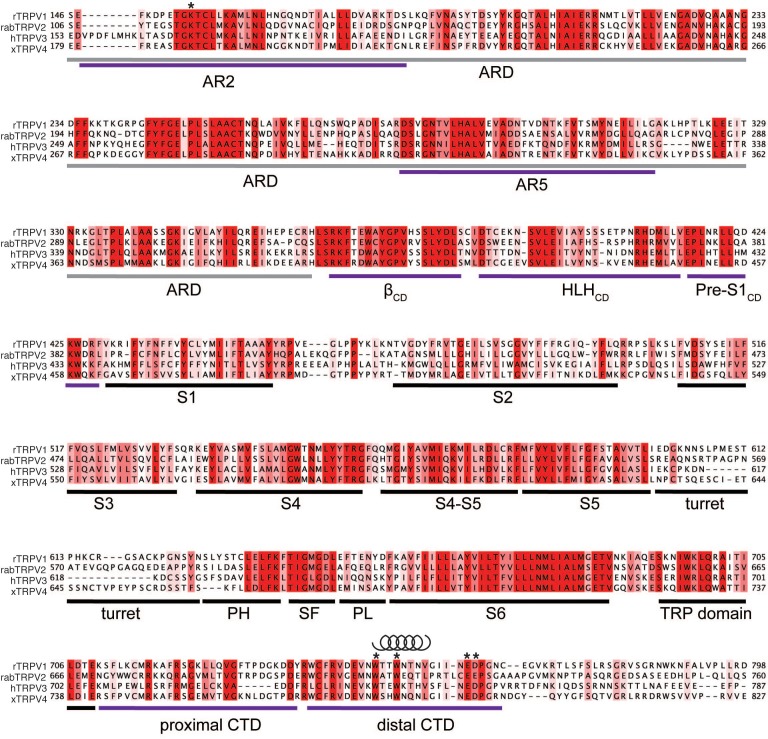
Sequence alignment of rat TRPV1 (rTRPV), rabbit TRPV2 (rabTRPV2), human TRPV3 (hTRPV3) and xenopus TRPV4 (xTRPV4). The alignment is colored by conservation and regions of interest are marked with purple lines. residues of interest are indicated with *. The distal CTD has a high helical propensity as calculated by PsiPred. Indicated by a helix cartoon. Abbreviations: ARD, ankyrin repeat domain; AR2, ankyrin repeat 2; AR5, ankyrin repeat 5; β_CD_, β-sheet of the coupling domain (CD); HLH _CD_, helix-loop-helix of the CD; Pre-S1 _CD,_ Pre-S1 of the CD; PH, pore helix; SF, selectivity filter; PL, pore loop.

## Discussion

**Figure 6.**
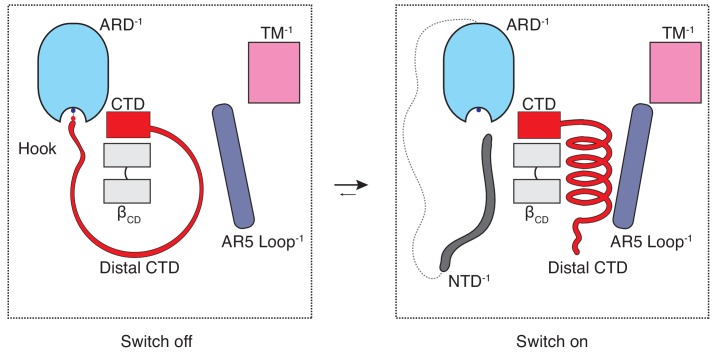
The CTD-mediated ‘switch’ gating mechanism. In the naive closed state (C_0_), the distal CTD is stretched around the β_CD_ via a salt bridge ‘hook’ interaction with the ARD and the switch is ‘off’. When the CTD is ‘unhooked’ from the ARD, it undergoes a coil-to-helix transition (the switch turns ‘on’) which leads to a conformational change in the loop of AR5 and consequently to increased coupling between the cytoplasmic and transmembrane domains, which is prerequisite for channel opening (C_1_).

The majority of the thermoTRPV channel structure resides in the cytoplasm and it has long been known that mutations in the cytoplasmic domains of these channels profoundly affect channel function. However, the role of these domains in thermoTRPV gating has been unclear since recent mechanistic studies of thermoTRPV channels have largely focused on the transmembrane domains. To the best of our knowledge, this study offers an unprecedented mechanistic insight into the involvement of the cytoplasmic domains in thermoTRPV gating as well as a potential explanation for the subtype specific ligand- and heat-dependent sensitization and activation phenotypes. The combination of our structural data and functional experiments provides evidence that hysteresis, sensitization, and activation of TRPV3 involves increased coupling between structural elements from the cytoplasmic (ARD and CD) and the transmembrane domains (TRP and S6) is triggered by a coil-to-helix transition in the distal CTD. The distal CTD appears to play a critical role akin to a binary switch in this coupling process. When the channel is in the naive closed state (C_0_) the distal CTD adopts a coil (‘off’) conformation which is stretched around β_CD_ via a salt-bridge ‘hook’ between K169 in the ARD and E751 and D752 in the CTD. When the salt-bridge is disrupted and the distal CTD ‘unhooked’, the coil readily springs into a helical (‘on’) conformation, engages the loop of AR5, instigating a sequence of events that increase coupling in the sensitized C_1_ state that is prerequisite for opening (Figure 6). We propose that this switch-like process involving a coil-to-helix secondary structure transition of the distal CTD is responsible, at least in part, for the use-dependent irreversible sensitization of TRPV3. In agreement with our previous report (Zubcevic et al., 2018b), the α-to-π helical transition in the pore lining S6 helix also forms a part of the use-dependent sensitization trajectory. We posit that the α-to-π helical transition in S6 constitutes an early and integral conformational transition leading to sensitization of the wild-type TRPV3 because it could be captured upon chemical sensitization of the channel, unlike the coil-to-helix transition (Zubcevic et al., 2018b). Because the coil-to-helix transition involves large rearrangements in secondary structure and changes in inter-protomer interactions, it is likely that it needs to surmount a larger energy barrier than the α-to-π transition in S6.

Our structural and functional analyses suggest that the unique cytoplasmic inter-protomer interface, which exists in all thermoTRPV channels, is a determinant of their distinct physiological and biophysical properties with the distal CTD acting as a switch between functional states in channel gating. The cytoplasmic inter-protomer interface and its coupling through to the transmembrane domains via the distal CTD, the ARD, CD, the TRP domain and S6 is conserved in thermoTRPV channels (Figure 4—figure supplement 5, Figure 5—figure supplement 1). The differences in the initial conformational state of the distal CTD from different thermoTRPV subtypes appear to correlate with their distinct sensitization and activation properties. The coil CTD, or ‘off’, conformation in the sensitizing TRPV2 and TRPV3 keeps channels in conformations associated with the closed C_0_ state. By contrast, the CTD of the non-sensitizing TRPV1 is consistent with a helical ‘on’ conformation associated with the primed C_1_ closed state. This proposes that the coupling network between the cytoplasmic and transmembrane domains can be modulated in a subtype-specific manner. The concept of channel gating via the cytoplasmic switch provides a framework for further examination of both ligand and heat dependent gating of thermoTRPV channels, which could be extended to other ion channel families.

## Materials and methods

**Key resources table:** 

Reagent type (species) or resource	Designation	Source or reference	Identifiers	Additional information
Cell line (*E. coli*)	DH10Bac	ThermoFisher Scientific	10361012	
Cell line (*Spodoptera frugiperda*)	Sf9	ATCC	CRL-1711	RRID:CVCL_0549
Cell line (*Homo sapiens*)	HEK293T	ATCC	CRL-11268; Lot Number 62312975	RRID:CVCL_0063
Cell media component	Dulbecco’s Modified Eagle’s Medium (DMEM) - low glucose	Gibco	11885–084	
Cell media component	Heat Inactivated Fetal Bovine Serum	Gibco	10082–139	
Cell media component	Anti-Anti (Antibiotic-Antiycotic)	Gibco	15240–062	
Recombinant DNA reagent	human TRPV3	Genscript	Pubmed Gene ID: 162514	
Recombinant DNA reagent	Bac-to-Bac Baculovirus Expression System	ThermoFisher Scientific	10359016	
Recombinant DNA reagent	FuGene6	Promega	E2691	
Chemical compound, drug	*n*-dodecyl-β-d- maltopyranoside(DDM)	Anatrace	D310	
Chemical compound, drug	Cholesteryl Hemisuccinate	Anatrace	CH210	
Chemical compound, drug	PMAL-C8	Anatrace	P5008	
Chemical compound, drug	TRIS	Fisher Scientific	BP152	
Chemical compound, drug	NaCl	Fisher Scientific	S271	
Chemical compound, drug	CaCl_2_	Fisher Scientific	C70	
Chemical compound, drug	KCl	Sigma Aldrich	P9333	
Chemical compound, drug	MgCl_2_	Sigma Aldrich	M8266	
Chemical compound, drug	4-(2-hydroxyethyl)−1- piperazineethanesulfonic acid (HEPES)	Sigma Aldrich	H3375	
Chemical compound, drug	NaOH	Sigma Aldrich	S5881	
Chemical compound, drug	CsCl	Sigma Aldrich	C3139	
Chemical compound, drug	Ethylene glycol-bis (2-aminoethylether)- N,N,N′,N′-tetraacetic acid (EGTA)	Sigma Aldrich	E4378	
Chemical compound, drug	CsOH solution	Sigma Aldrich	232041	
Chemical compound, drug	2-Aminoethyl diphenylborinate (2-APB)	Sigma Aldrich	D9754	
Chemical compound, drug	D-Camphor	Sigma Aldrich	W223018	
Chemical compound, drug	Dimethyl sulfoxide (DMSO)	Sigma Aldrich	D2650	
Chemical compound, drug	leupeptin	GoldBio	L-010	
Chemical compound, drug	pepstatin	GoldBio	P-020	
Chemical compound, drug	aprotinin	GoldBio	A-655	
Chemical compound, drug	DNase I	GoldBio	D-301	
Chemical compound, drug	β-mercapto ethanol	Sigma Aldrich	M3148	
Chemical compound, drug	PMSF	Sigma Aldrich	P7626	
Chemical compound, drug	anti-FLAG resin	Sigma Aldrich	A4596	
Chemical compound, drug	Bio-Beads SM-2	BioRad	152–8920	
Chemical compound, drug	1-palmitoyl-2-oleoyl -*sn*-glycero-3-phosphocholine (POPC)	Avanti Polar Lipids	850457C	
Chemical compound, drug	1-palmitoyl-2-oleoyl -*sn*-glycero-3- phosphoethanolamine (POPE)	Avanti Polar Lipids	850757C	
Chemical compound, drug	1-palmitoyl-2-oleoyl -*sn*-glycero-3-phospho- (1'-*rac*-glycerol) (POPG)	Avanti Polar Lipids	840457C	
Software, algorithm	MotionCor2	Zheng et al., 2017	http://msg.ucsf.edu/em/software/motioncor2.html	RRID:SCR_016499
Software, algorithm	GCTF	Zhang, 2016	https://www.mrc-lmb.cam.ac.uk/kzhang/	RRID:SCR_016500
Software, algorithm	RELION 3.0	Zivanov et al., 2018	https://www2.mrc-lmb.cam.ac.uk/relion/	RRID:SCR_016274
Software, algorithm	Coot	Emsley and Cowtan, 2004	https://www2.mrc-lmb.cam.ac.uk/personal/pemsley/coot/	RRID:SCR_014222
Software, algorithm	Phenix	Adams et al., 2010	http://phenix-online.org/	RRID:SCR_014224
Software, algorithm	Molprobity	Chen et al., 2010	http://molprobity.biochem.duke.edu/index.php	RRID:SCR_014226
Software, algorithm	UCSF Chimera	Pettersen et al., 2004	https://www.cgl.ucsf.edu/chimera/	RRID:SCR_004097
Software, algorithm	Pymol	Shrödinger LLC	https://pymol.org/2/	RRID:SCR_000305
Software, algorithm	pClamp10	Molecular Devices		RRID:SCR_011323
Software, algorithm	OriginPro 2016	OriginLab Corp.		RRID:SCR_014212
Software, algorithm	Microsoft Excel 2010	Microsoft		RRID:SCR_016137
other	Whatman No. one filter paper	Sigma Aldrich	WHA1001325	
Other	UltrAuFoil R1.2/1.3 300-mesh grid	Electron Microscopy Sciences	Q350AR13A	
Other	Cryo-electron microscopy structure of the human TRPV3 channel	Zubcevic et al., 2018a	PDB ID 6MHO	Zubcevic et al., 2018b
Other	Cryo-electron microscopy structure of the human TRPV3 channel	Zubcevic et al., 2018a	EMDB ID EMD-9115	Zubcevic et al., 2018b

### Expression and purification of human TRPV3

A full-length human TRPV3 construct, containing mutations T96A (Zubcevic et al., 2018b) and K169A was cloned into a pFastBac vector in frame with a FLAG affinity tag, and baculovirus was produced according to manufacturers’ protocol (Invitrogen, Bac-to-Bac). Sf9 insect cells (ATCC), infected with baculovirus at a density of 1.3 × 10^6^ cells ml^−1^, were grown for 72 hr at 27° C in an orbital shaker. Cell pellets were collected and resuspended in buffer A (50 mM TRIS pH 8, 150 mM NaCl, 1 μg ml^−1^ leupeptin, 1.5 μg ml^−1^ pepstatin, 0.84 μg ml^−1^ aprotinin, 0.3 mM PMSF, 14.3 mM β-mercaptoethanol, and DNAseI) before lysis by sonication (3 × 30 pulses). Lysed cells were solubilized in 40 mM dodecyl β-maltoside (DDM, Anatrace) and 4 mM Cholesteryl Hemisuccinate Tris salt (CHS, Anatrace) at 4° C for 1 hr. Insoluble material was removed by centrifugation (8,000 *g*, 30 min), and anti-FLAG resin was added to the supernatant for 1 hr at 4° C. Following binding, the anti-FLAG resin was transferred to a Biorad column at 4° C and washed with 10 column volumes buffer B (50 mM TRIS pH8, 150 mM NaCl, 1 mM DDM, 0.1 mM CHS, 10 mM DTT) and the protein eluted in buffer C (50 mM TRIS pH 8, 150 mM NaCl, 1 mM DDM, 0.1 mM CHS, 0.1 mg ml^−1^ 3:1:1 1-palmitoyl-2-oleoyl-*sn*-glycero-3-phosphocholine (POPC), 1-palmitoyl-2-oleoyl-*sn*-glycero-3-phosphoethanolamine (POPE), 1-palmitoyl-2-oleoyl-*sn*-glycero-3-phospho-(1'-*rac*-glycerol) (POPG), 10 mM DTT, 10 mg ml^−1^ FLAG peptide). Size exclusion chromatography was performed and the protein peak collected and mixed with Poly (Maleic Anhydride-alt-1-Decene) substituted with 3-(Dimethylamino) Propylamine (PMAL-C8, Anatrace) (1:10 w/w ratio) and incubated overnight at 4° C with gentle agitation. Detergent was removed with Bio-Beads SM-2 (15 mg ml^−1^) for 1 hr at 4° C. The reconstituted protein was purified on a Superose 6 column at 4° C in buffer D (50 mM Tris pH8, 150 mM NaCl). Following size exclusion, the protein peak was collected and concentrated to 2–2.5 mg ml^−1^. For the TRPV3_K169A 2-APB_ sample, the protein was incubated with 1 mM 2-APB for ~3.5 min before blotting.

### Cryo-EM sample preparation

Cryo-EM grid preparation was performed similarly for each TRPV3 K169A specimen. 3 μl sample was dispensed on a freshly glow discharged (30 s) UltrAuFoil R1.2/1.3 300-mesh grid (Electron Microscopy Services), blotted for 3 s with Whatman No. one filter paper using the Leica EM GP2 Automatic Plunge Freezer at 23° C and >85% humidity and plunge-frozen in liquid ethane cooled by liquid nitrogen.

### Cryo-EM data collection

Data for TRPV3_K169A_ and TRPV3_K169A 2-APB_ was collected using the Titan Krios transmission electron microscope (TEM) operating at 300 keV using Gatan K2 Direct Electron Detector and a Falcon III Direct Electron Detector operating in counting mode, respectively. The nominal magnification used for the TRPV2_K169A_ sample was 130,000x corresponding to a physical pixel size of 1.06 Å/pixel. For the TRPV2_K169A 2-APB_, the nominal magnification was 75,000x corresponding to a physical pixel size of 1.08 Å/pixel. For the TRPV3_K169A_, 2385 movies (40 frames/movie) were collected using a 10 s exposure with an exposure rate of ~4.5 e^-^/pixel/s, resulting in a total exposure of 40 e^-^/Å (Caterina et al., 1997) and a nominal defocus range from −1.0 µm to −2.5 µm. For TRPV3_K169A 2-APB_, 1984 movies were collected (30 frames/movie) with 60 s exposure and exposure rate of ~0.8 e^-^/pixel/s. The total exposure was of 42 e^-^/Å (Caterina et al., 1997) and a nominal defocus range from −1.25 µm to −3.0 µm.

### Reconstruction and refinement

*TRPV3_K169A_* MotionCor2 (Zheng et al., 2017) was used to perform motion correction and dose-weighting on 2385 movies. Summed unweighted images were used for CTF determination using GCTF (Zhang, 2016). After motion correction and CTF determination, the dataset was pruned by removing micrographs which contained Figure of Merit (FoM) values of <0.05 and Astigmatism values > 1700. A set of 1596 particles was picked manually and subjected to reference-free 2D classification (k = 12, T = 2) which subsequently served as a template for automatic particle picking from the entire dataset. A stack of 452,388 particles were picked (binned 4 × 4 (4.24 Å/pixel, 64 pixel box size)) and subjected to reference-free 2-D classification (k = 45, T = 2) in RELION 3.0 (Zivanov et al., 2018). Classes displaying the most well-defined secondary structure features were selected (441,547 particles) and used in 3D refinement with no symmetry imposed (C1) and with the previously determined map for apo human TRPV3 (EMD-9115) filtered to 30 Å as a reference model. This resulted in an 8.7 Å 3D reconstruction, which was then used for re-extraction and re-centering of 1 × 1 binned particles (1.06 Å/pixel, 256 pixel box size). 3D classification (k = 6, T = 8) without imposed symmetry (C1) was performed on these particles, using a soft mask calculated from the full molecule. Class 4 (95,184 particles) possessed the most well-defined secondary structure elements and was chosen for further analysis. 3D auto-refinement of class four without symmetry imposed (C1) yielded a 4.6 Å 3D reconstructions. The particles were then subjected to Bayesian polishing as implemented in RELION 3.0. The shiny particles were input into 3D auto-refinement with a soft mask no imposed symmetry (C1), resulting in a 4.3 Å reconstruction. These particles were subjected to CTF refinement, followed by another round of 3D auto-refinement in C1 symmetry resulting in a 4.37 Å map. Visual inspection of the volume revealed the presence of four-fold symmetry, and therefore 3D auto-refinement was repeated with C4 symmetry imposed, resulting in the final 4.1 Å map.

*TRPV3_K169A 2-APB_* 1984 movies were subjected to motion correction and dose-weighing using MotionCor2. The unweighted and summed images were used for CTF determination using GCTF. Micrographs with a Figure of Merit (FoM) values of <0.15 and Astigmatism values of >200 were removed. A set of 2099 particles was picked manually and subjected to reference-free 2D classification (k = 12, T = 2) which were used as a template for automatic particle picking from the entire dataset. This resulted in a stack of 1,174,521 particles (binned 4 × 4 (4.32 Å/pixel, 64 pixel box size)) and subjected to reference-free 2-D classification (k = 75, T = 2) in RELION 3.0 (Zivanov et al., 2018). Classes with the most well-defined structural features were picked (636,742 particles) and extracted (binned 1 × 1 (1.08 Å/pixel, 256 pixel box size)) before being subjected to 3D auto-refinement using the map of human apo TRPV3 (EMD-9115) filtered to 30 Å as a reference and with no symmetry imposed (C1), resulting in a 5.3 Å reconstruction. This was then subjected to 3D classification (k = 6, T = 8) which included particle alignment and with no symmetry imposed. Class 4 (136,814 particles) was selected and subjected to 3D auto-refinement (C1), yielding a 3.9 Å reconstruction. Further 3D classification (k = 2, T = 8) using a soft mask and without alignment or imposed symmetry separated a fraction of bad particles (57,808 particles) leaving a stack of 79,006 particles which were subjected to another round of 3D auto-refinement (C1). The resulting volume revealed four-fold symmetry and the 3D auto-refinement was therefore repeated with C4 symmetry imposed, yielding a 3.6 Å reconstruction. These particles were then subjected to Bayesian polishing and CTF refinement, resulting in a final reconstruction resolved to 3.59 Å. All resolution estimates were based on the gold-standard FSC 0.143 criterion (Scheres and Chen, 2012; Chen et al., 2013).

### Model building

The TRPV3_K169A_ and TRPV3_K169A 2-APB_ models were built directly into the cryo-EM electron density using the previously determined structure of the human TRPV3 in the apo form (PDB 6MHO) as a template. The models were first refined in real space in Coot (Emsley and Cowtan, 2004) and subsequently subjected to automated real space refinement using phenix.real_space_refine as implemented in the Phenix suite (Adams et al., 2010). The refinement was performed using global minimization and rigid body, with tight ideal geometry and secondary structure restraints. The refinement process was guided by the Molprobity server (http://molprobity.biochem.duke.edu/) (Chen et al., 2010). Analysis and structure illustrations were performed using Pymol (The PyMOL Molecular Graphics System, Version 2.0) and UCSF Chimera (Pettersen et al., 2004).

### Model comparisons and analysis

All structural alignments and measurements were performed using Pymol and UCSF Chimera. The correlation of the fit of the coil vs. the helix distal CTD into the mTRPV3 cryo-EM map (EMD-8921) was calculated as follows: the EMD-8921 map was segmented using the *Segment map* tool, as implemented in UCSF Chimera, and the density corresponding to the distal CTD was isolated and saved as a map file (Map_CTD_). The coil CTD (PDB 6DVZ) and the helical CTD (TRPV3_K169A 2-APB_) were isolated and saved as individual pdb files (CTD_coil_ and CTD_helix_) and placed in the Map_CTD._ Using the *molmap* function in UCSF Chimera, simulated maps were generated for CTD_coil_ and CTD_helix_ at the same resolution as Map_CTD_. Correlation was calculated between the CTD_coil_/Map_CTD_, and CTD_helix/_Map_CTD_ using the *measure correlation* function in UCSF Chimera.

### Cell lines

HEK293T cells were purchased from ATCC with authentication records. Additional authentication was not performed prior to this study. Cells tested negative for mycoplasma contamination.

### Electrophysiology

HEK293T cells (62312975 – ATCC) were grown in DMEM supplemented with 10% FBS (Gibco), 1% penicillin/streptomycin (Gibco) and were sustained at 37°C in 5% CO_2_. Cells between passage 10–30 grown in 40 mm wells were transiently transfected at ~50% confluency with plasmids encoding for either WT, K169A, E751A, W739A, W742A, K743A, R487A, R487W, E501G, Y540W, Y565A, H426A TRPV3 and green fluorescent protein (GFP) using FuGene6 (Promega). ~24 hr after transfection, cells were reseeded onto 12 mm round glass coverslips (Fisher) in 20 mm wells and used after 12–24 hr for electrophysiological measurements.

Voltage-clamp recording were performed in the whole-cell patch-clamp configuration with electrodes pulled from borosilicate glass capillaries (Sutter Instruments) with a final resistance of 2–5 MΩ. Electrodes were filled with an intracellular solution containing (in mM) 150 CsCl, 1 MgCl_2_, 10 HEPES, 5 EGTA, and adjusted to pH 7.2 (CsOH). Glass coverslips with adherent transfected cells were placed into an open bath chamber (RC-26G, Warner Instruments) with an extracellular wash solution containing (in mM) 140 NaCl, 5 KCl, 1 MgCl_2_, 10 HEPES at pH 7.4 (NaOH). Extracellular wash solutions were used to make solutions containing; 2-aminoethoxydiphenyl borate (2-APB) (Sigma) (prepared daily from DMSO stocks (1 M) stored at −80° C; final DMSO 0.03%), ruthenium red (RuR) (Sigma) (prepared daily from water stock (10 mM) stored at −80° C; final DMSO 0.03%), and D-camphor (Sigma) (prepared daily as previously described [Xu et al., 2005] from DMSO stocks (2 M) stored at −80° C). Solutions were focally applied to patched calls with a pressurized perfusion system (BPS-8, ALA Scientific Instruments). Current responses were low-pass filtered at 2 kHz (Axopatch 200B), digitally sampled at 5–10 kHz (Digidata 1440A), converted to digital files in Clampex10.7 (Molecular Devices) and stored on an external hard drive for offline analyses (Clampfit10.7, Molecular Devices; Excel 2010, Microsoft Office; OriginPro 2016, OrginLab Corp).

2-APB sensitization experiments were performed as previously described (Zubcevic et al., 2018b). Briefly, a 30 s continuously repeating protocol (holding potential of +60 mV) was used in which cells were first perfused with extracellular wash for 1 s, followed by a 15 s application of 30 μM 2-APB extracellular solution, preceded by 14 s of wash solution. Recordings that displayed little to no current response after ~25–30 rounds of 2-APB stimulation were stopped.

The remaining residual sensitized current following washout of 30 μM 2-APB was measured immediately following the 2-APB sensitization protocol. Sensitized cells underwent a protocol (holding potential of +60 mV) in which extracellular wash was perfused for 10 s, followed by 15 s of 30 μM 2-APB, then washed again for 30 s, proceeded by application of 50 μM RuR for 10 s. The RuR-sensitive residual current was calculated by the difference between the second wash (I_wash2_) and RuR (I_RuR_) current amplitude (I_wash2_ – I_RuR_) and is reported as current density.

Spontaneous basal channel activity was assessed prior to 2-APB-induced sensitization. After achieving the whole-cell configuration, cells were held at 0 mV for 5 s and underwent a voltage step +60 mV for 10 s. 5 s after the voltage step to +60 mV, the cell was perfused with 50 μM RuR for the last 5 s of the protocol. RuR sensitive basal currents were calculated as the difference between the measured wash (I_wash_) and RuR (I_RuR_) current (I_wash_ – I_RuR_) amplitudes at +60 mV and are reported as current density.

Whole-cell voltage step protocol from −120 to +200 mV (Δ 20 mV, 500 ms) was immediately followed by a −160 mV post-test pulse for 200 ms with a holding potential of −60 mV. The peak tail current amplitudes from the −160 mV post-test pulse were used to calculate the corresponding conductance amplitude for each voltage step. Since the tail currents from the +200 mV step did not result in saturating tail current amplitudes, G/Gmax curves were not constructed and the data was plotted as the conductance amplitudes versus the step voltage.

Hysteresis and sensitization were assessed by measuring changes in the EC_50_ of 2-APB sensitivity following consecutive dose-response rounds. Cells underwent a recording protocol (holding potential of +60 mV) that started with a 1 s wash, followed by 15 s application of a single concentration of 2-APB (3, 10, 30, 50, 100 or 300 μM; final DMSO 0.03%), followed by 15 s of wash and was repeated for each concertation, in order from lowest to highest concentration for each dose-response round. Cells were subjected to three consecutive rounds of this dose-response protocol.

2-APB binding site testing experiments were performed with a continuously repeating voltage ramp protocol (holding potential 0 mV, 400 ms voltage ramp from −60 to +60 mV) elicited every 5 s. Cells were first perfused for 30 s with 30 μM 2-APB, followed by 300 μM 2-APB, and finally 10 mM camphor, with a 30 s wash between each application of ligand. The ratio between both 30 and 300 μM 2-APB to the camphor current response at +60 mV was calculated. Leak was assessed at the end of the recording with application of 50 μM RuR.

Sensitization was characterized by the ratio of the response to 2-APB during the first (I_0_) and maximum current (I_max_) response (I_max_/I_0_) calculated as the mean from each biologically independent experiment.

*Data Analysis* 2-APB sensitization parameters were evaluated by the stimulation dependent increase in the peak current amplitude measured at the end of each 2-APB exposure as previously described (Zubcevic et al., 2018b). Briefly, peak current amplitudes (*I*) from each individual stimulation was normalized to the maximum peak current (*I_max_*) amplitude, and the fractional current (*I*/*I_max_*) of each stimulation was plotted by stimulation number for each individual recording.

The relative extent of sensitization was characterized by the increase in current amplitude obtained during the first (*I_0_*) and maximum current (*I_max_*) stimulation (*I_max_*/*I_0_*) and calculated as the mean from each biologically independent experiment. The first five 2-APB stimulations per recordings was also plotted as current density for comparison between conditions.

2-APB dose-response data for each individual round were fit with the Hill equation from biologically independent experiments. The average EC_50_ values from each fit was calculated for each dose-response. The averaged normalized current response from each 2-APB concertation (3, 10, 30, 50, 100, 300 μM) per round were averaged and fit with the Hill equation to calculate the corresponding EC_50_ and Hill coefficient (*n*_H_) for each construct tested.

## Data Availability

Cryo-EM data and structural models are deposited in the EMDB and RCSB, respectively with the following codes: EMD-20192, PDB: 6OT2 and EMD-20194, PDB: 6OT5 The following datasets were generated: ZubcevicLWilliamF BorschelAllenL HsuMarioJ BorgniaLeeS-Y2019Structure of the TRPV3 K169A sensitized mutant in the presence of 2-APB at 3.6 A resolutionElectron Microscopy Data BankEMD-20194 ZubcevicLWilliamF BorschelAllenL HsuMarioJ BorgniaLeeS-Y2019Structure of the TRPV3 K169A sensitized mutant in the presence of 2-APB at 3.6 A resolutionProtien Data Bank6OT5 ZubcevicLWilliamF BorschelAllenL HsuMarioJ BorgniaLeeS-Y2019Structure of the TRPV3 K169A sensitized mutant in apo form at 4.1 A resolutionElectron Microscopy Data BankEMD-20192 ZubcevicLWilliamF BorschelAllenL HsuMarioJ BorgniaLeeS-Y2019Structure of the TRPV3 K169A sensitized mutant in apo form at 4.1 A resolutionProtein Data Bank6OT2

## Additional files

Transparent reporting form

## References

[bib1] Adams PD, Afonine PV, Bunkóczi G, Chen VB, Davis IW, Echols N, Headd JJ, Hung LW, Kapral GJ, Grosse-Kunstleve RW, McCoy AJ, Moriarty NW, Oeffner R, Read RJ, Richardson DC, Richardson JS, Terwilliger TC, Zwart PH (2010). PHENIX: a comprehensive Python-based system for macromolecular structure solution. Acta Crystallographica Section D Biological Crystallography.

[bib2] Bang S, Yoo S, Yang TJ, Cho H, Hwang SW (2010). Farnesyl pyrophosphate is a novel pain-producing molecule via specific activation of TRPV3. Journal of Biological Chemistry.

[bib3] Brauchi S, Orta G, Salazar M, Rosenmann E, Latorre R (2006). A hot-sensing cold receptor: C-terminal domain determines thermosensation in transient receptor potential channels. Journal of Neuroscience.

[bib4] Cao E, Liao M, Cheng Y, Julius D (2013). TRPV1 structures in distinct conformations reveal activation mechanisms. Nature.

[bib5] Caterina MJ, Schumacher MA, Tominaga M, Rosen TA, Levine JD, Julius D (1997). The capsaicin receptor: a heat-activated ion channel in the pain pathway. Nature.

[bib6] Caterina MJ, Rosen TA, Tominaga M, Brake AJ, Julius D (1999). A capsaicin-receptor homologue with a high threshold for noxious heat. Nature.

[bib7] Caterina MJ, Leffler A, Malmberg AB, Martin WJ, Trafton J, Petersen-Zeitz KR, Koltzenburg M, Basbaum AI, Julius D (2000). Impaired nociception and pain sensation in mice lacking the capsaicin receptor. Science.

[bib8] Chen VB, Arendall WB, Headd JJ, Keedy DA, Immormino RM, Kapral GJ, Murray LW, Richardson JS, Richardson DC (2010). MolProbity: all-atom structure validation for macromolecular crystallography. Acta Crystallographica Section D Biological Crystallography.

[bib9] Chen S, McMullan G, Faruqi AR, Murshudov GN, Short JM, Scheres SH, Henderson R (2013). High-resolution noise substitution to measure overfitting and validate resolution in 3D structure determination by single particle electron cryomicroscopy. Ultramicroscopy.

[bib10] Deng Z, Paknejad N, Maksaev G, Sala-Rabanal M, Nichols CG, Hite RK, Yuan P (2018). Cryo-EM and X-ray structures of TRPV4 reveal insight into ion permeation and gating mechanisms. Nature Structural & Molecular Biology.

[bib11] Emsley P, Cowtan K (2004). Coot: model-building tools for molecular graphics. Acta Crystallographica. Section D, Biological Crystallography.

[bib12] Gavva NR, Treanor JJ, Garami A, Fang L, Surapaneni S, Akrami A, Alvarez F, Bak A, Darling M, Gore A, Jang GR, Kesslak JP, Ni L, Norman MH, Palluconi G, Rose MJ, Salfi M, Tan E, Romanovsky AA, Banfield C, Davar G (2008). Pharmacological blockade of the vanilloid receptor TRPV1 elicits marked hyperthermia in humans. Pain.

[bib13] Gopinath P, Wan E, Holdcroft A, Facer P, Davis JB, Smith GD, Bountra C, Anand P (2005). Increased capsaicin receptor TRPV1 in skin nerve fibres and related vanilloid receptors TRPV3 and TRPV4 in keratinocytes in human breast pain. BMC Women's Health.

[bib14] Güler AD, Lee H, Iida T, Shimizu I, Tominaga M, Caterina M (2002). Heat-Evoked activation of the ion channel, TRPV4. The Journal of Neuroscience.

[bib15] Gunthorpe MJ, Harries MH, Prinjha RK, Davis JB, Randall A (2000). Voltage- and time-dependent properties of the recombinant rat vanilloid receptor (rVR1). The Journal of Physiology.

[bib16] Gunthorpe MJ, Benham CD, Randall A, Davis JB (2002). The diversity in the vanilloid (TRPV) receptor family of ion channels. Trends in Pharmacological Sciences.

[bib17] Hu H, Grandl J, Bandell M, Petrus M, Patapoutian A (2009). Two amino acid residues determine 2-APB sensitivity of the ion channels TRPV3 and TRPV4. PNAS.

[bib18] Huang SM, Chung MK (2013). Targeting TRPV3 for the development of novel analgesics. The Open Pain Journal.

[bib19] Huynh KW, Cohen MR, Jiang J, Samanta A, Lodowski DT, Zhou ZH, Moiseenkova-Bell VY (2016). Structure of the full-length TRPV2 channel by cryo-EM. Nature Communications.

[bib20] Joseph J, Wang S, Lee J, Ro JY, Chung MK (2013). Carboxyl-terminal domain of transient receptor potential vanilloid 1 contains distinct segments differentially involved in capsaicin- and heat-induced desensitization. Journal of Biological Chemistry.

[bib21] Landouré G, Zdebik AA, Martinez TL, Burnett BG, Stanescu HC, Inada H, Shi Y, Taye AA, Kong L, Munns CH, Choo SS, Phelps CB, Paudel R, Houlden H, Ludlow CL, Caterina MJ, Gaudet R, Kleta R, Fischbeck KH, Sumner CJ (2010). Mutations in TRPV4 cause Charcot-Marie-Tooth disease type 2C. Nature Genetics.

[bib22] Liao M, Cao E, Julius D, Cheng Y (2013). Structure of the TRPV1 ion channel determined by electron cryo-microscopy. Nature.

[bib23] Lishko PV, Procko E, Jin X, Phelps CB, Gaudet R (2007). The ankyrin repeats of TRPV1 bind multiple ligands and modulate channel sensitivity. Neuron.

[bib24] Liu B, Yao J, Zhu MX, Qin F (2011). Hysteresis of gating underlines sensitization of TRPV3 channels. The Journal of General Physiology.

[bib25] Liu B, Qin F (2016). Use dependence of heat sensitivity of vanilloid receptor TRPV2. Biophysical Journal.

[bib26] Liu B, Qin F (2017). Single-residue molecular switch for high-temperature dependence of vanilloid receptor TRPV3. PNAS.

[bib27] Marics I, Malapert P, Reynders A, Gaillard S, Moqrich A (2014). Acute heat-evoked temperature sensation is impaired but not abolished in mice lacking TRPV1 and TRPV3 channels. PLOS ONE.

[bib28] Moqrich A, Hwang SW, Earley TJ, Petrus MJ, Murray AN, Spencer KS, Andahazy M, Story GM, Patapoutian A (2005). Impaired thermosensation in mice lacking TRPV3, a heat and camphor sensor in the skin. Science.

[bib29] Patapoutian A (2005). TRP channels and thermosensation. Chemical Senses.

[bib30] Pettersen EF, Goddard TD, Huang CC, Couch GS, Greenblatt DM, Meng EC, Ferrin TE (2004). UCSF chimera--a visualization system for exploratory research and analysis. Journal of Computational Chemistry.

[bib31] Phelps CB, Wang RR, Choo SS, Gaudet R (2010). Differential regulation of TRPV1, TRPV3, and TRPV4 sensitivity through a conserved binding site on the ankyrin repeat domain. Journal of Biological Chemistry.

[bib32] Reilly RM, Kym PR (2011). Analgesic potential of TRPV3 antagonists. Current Topics in Medicinal Chemistry.

[bib33] Salazar H, Llorente I, Jara-Oseguera A, García-Villegas R, Munari M, Gordon SE, Islas LD, Rosenbaum T (2008). A single N-terminal cysteine in TRPV1 determines activation by pungent compounds from onion and garlic. Nature Neuroscience.

[bib34] Sánchez-Moreno A, Guevara-Hernández E, Contreras-Cervera R, Rangel-Yescas G, Ladrón-de-Guevara E, Rosenbaum T, Islas LD (2018). Irreversible temperature gating in trpv1 sheds light on channel activation. eLife.

[bib35] Scheres SH, Chen S (2012). Prevention of overfitting in cryo-EM structure determination. Nature Methods.

[bib36] Shi DJ, Ye S, Cao X, Zhang R, Wang K (2013). Crystal structure of the N-terminal ankyrin repeat domain of TRPV3 reveals unique conformation of finger 3 loop critical for channel function. Protein & Cell.

[bib37] Singh AK, McGoldrick LL, Sobolevsky AI (2018). Structure and gating mechanism of the transient receptor potential channel TRPV3. Nature Structural & Molecular Biology.

[bib38] Smith GD, Gunthorpe MJ, Kelsell RE, Hayes PD, Reilly P, Facer P, Wright JE, Jerman JC, Walhin JP, Ooi L, Egerton J, Charles KJ, Smart D, Randall AD, Anand P, Davis JB (2002). TRPV3 is a temperature-sensitive vanilloid receptor-like protein. Nature.

[bib39] Voets T, Droogmans G, Wissenbach U, Janssens A, Flockerzi V, Nilius B (2004). The principle of temperature-dependent gating in cold- and heat-sensitive TRP channels. Nature.

[bib40] Xu H, Ramsey IS, Kotecha SA, Moran MM, Chong JA, Lawson D, Ge P, Lilly J, Silos-Santiago I, Xie Y, DiStefano PS, Curtis R, Clapham DE (2002). TRPV3 is a calcium-permeable temperature-sensitive cation channel. Nature.

[bib41] Xu H, Blair NT, Clapham DE (2005). Camphor activates and strongly desensitizes the transient receptor potential vanilloid subtype 1 channel in a vanilloid-independent mechanism. Journal of Neuroscience.

[bib42] Yao J, Liu B, Qin F (2011). Modular thermal sensors in temperature-gated transient receptor potential (TRP) channels. PNAS.

[bib43] Zanou N, Mondin L, Fuster C, Seghers F, Dufour I, de Clippele M, Schakman O, Tajeddine N, Iwata Y, Wakabayashi S, Voets T, Allard B, Gailly P (2015). Osmosensation in TRPV2 dominant negative expressing skeletal muscle fibres. The Journal of Physiology.

[bib44] Zhang F, Hanson SM, Jara-Oseguera A, Krepkiy D, Bae C, Pearce LV, Blumberg PM, Newstead S, Swartz KJ (2016). Engineering vanilloid-sensitivity into the rat TRPV2 channel. eLife.

[bib45] Zhang K (2016). Gctf: real-time CTF determination and correction. Journal of Structural Biology.

[bib46] Zhang F, Swartz KJ, Jara-Oseguera A (2019). Conserved allosteric pathways for activation of TRPV3 revealed through engineering vanilloid-sensitivity. eLife.

[bib47] Zheng SQ, Palovcak E, Armache JP, Verba KA, Cheng Y, Agard DA (2017). MotionCor2: anisotropic correction of beam-induced motion for improved cryo-electron microscopy. Nature Methods.

[bib48] Zivanov J, Nakane T, Forsberg BO, Kimanius D, Hagen WJ, Lindahl E, Scheres SH (2018). New tools for automated high-resolution cryo-EM structure determination in RELION-3. eLife.

[bib49] Zubcevic L, Herzik MA, Chung BC, Liu Z, Lander GC, Lee SY (2016). Cryo-electron microscopy structure of the TRPV2 ion channel. Nature Structural & Molecular Biology.

[bib50] Zubcevic L, Le S, Yang H, Lee SY (2018a). Conformational plasticity in the selectivity filter of the TRPV2 ion channel. Nature Structural & Molecular Biology.

[bib51] Zubcevic L, Herzik MA, Wu M, Borschel WF, Hirschi M, Song AS, Lander GC, Lee SY (2018b). Conformational ensemble of the human TRPV3 ion channel. Nature Communications.

