# Supplementary material for: Regulatory switch at the cytoplasmic interface controls TRPV channel gating
Source: eLife. 2019 May 9;8:e47746. doi: 10.7554/eLife.47746 (PMC6538378; doi:10.7554/eLife.47746)
Supplement: Transparent reporting form [file elife-47746-transrepform.docx]

***eLife’s* transparent reporting form**

We encourage authors to provide detailed information *within their submission* to facilitate the interpretation and replication of experiments. Authors can upload supporting documentation to indicate the use of appropriate reporting guidelines for health-related research (see [EQUATOR Network](http://www.equator-network.org/%20)), life science research (see the [BioSharing Information Resource](https://biosharing.org/)), or the [ARRIVE guidelines](http://www.plosbiology.org/article/info:doi/10.1371/journal.pbio.1000412) for reporting work involving animal research. Where applicable, authors should refer to any relevant reporting standards documents in this form.

If you have any questions, please consult our Journal Policies and/or contact us: [editorial@elifesciences.org](mailto:editorial@elifesciences.org).

**Sample-size estimation**

- You should state whether an appropriate sample size was computed when the study was being designed
- You should state the statistical method of sample size computation and any required assumptions
- If no explicit power analysis was used, you should describe how you decided what sample (replicate) size (number) to use

Please outline where this information can be found within the submission (e.g., sections or figure legends), or explain why this information doesn’t apply to your submission:

In the structural aspects of this study, we did not use statistical methods to predetermine the sample size. In the electrophysiological aspects of this study, statistical methods were not used to determine sample size.

**Replicates**

- You should report how often each experiment was performed
- You should include a definition of biological versus technical replication
- The data obtained should be provided and sufficient information should be provided to indicate the number of independent biological and/or technical replicates
- If you encountered any outliers, you should describe how these were handled
- Criteria for exclusion/inclusion of data should be clearly stated
- High-throughput sequence data should be uploaded before submission, with a private link for reviewers provided (these are available from both GEO and ArrayExpress)

Please outline where this information can be found within the submission (e.g., sections or figure legends), or explain why this information doesn’t apply to your submission:

In the structural aspects of this study, it is not applicable as structure determination is not replicated as cryo-EM maps represent the average of many replicas of the molecule.

In the electrophysiological aspects of this study, sample size is included in the figure legends. All data points for each replicate are provided in the figures as individual data points along with the mean ± S.E.M.

Patch-clamp recordings were assessed for leak following the end of the recording by the decrease in current with a blocker. Patches with large blocker insensitive currents (>25 pF/pA) were excluded from analyses.

**Statistical reporting**

- Statistical analysis methods should be described and justified
- Raw data should be presented in figures whenever informative to do so (typically when N per group is less than 10)
- For each experiment, you should identify the statistical tests used, exact values of N, definitions of center, methods of multiple test correction, and dispersion and precision measures (e.g., mean, median, SD, SEM, confidence intervals; and, for the major substantive results, a measure of effect size (e.g., Pearson's r, Cohen's d)
- Report exact p-values wherever possible alongside the summary statistics and 95% confidence intervals. These should be reported for all key questions and not only when the p-value is less than 0.05.

Please outline where this information can be found within the submission (e.g., sections or figure legends), or explain why this information doesn’t apply to your submission:

The cryo-EM map and atomic model statistics are listed in Table 1. Statistical analysis was not used for electrophysiological data is represented as a single data points for individual recordings and as mean ± S.E.M

(For large datasets, or papers with a very large number of statistical tests, you may upload a single table file with tests, Ns, etc., with reference to sections in the manuscript.)

**Group allocation**

- Indicate how samples were allocated into experimental groups (in the case of clinical studies, please specify allocation to treatment method); if randomization was used, please also state if restricted randomization was applied
- Indicate if masking was used during group allocation, data collection and/or data analysis

Please outline where this information can be found within the submission (e.g., sections or figure legends), or explain why this information doesn’t apply to your submission:

No premeditated or manual allocation of samples into experimental groups was performed. Random allocation of particles into half-maps was performed by RELION, as a part of the standard validation procedure. This is described in the Methods section. Not applicable for electrophysiological recordings, group allocation was not used.

**Additional data files (“source data”)**

- We encourage you to upload relevant additional data files, such as numerical data that are represented as a graph in a figure, or as a summary table
- Where provided, these should be in the most useful format, and they can be uploaded as “Source data” files linked to a main figure or table
- Include model definition files including the full list of parameters used
- Include code used for data analysis (e.g., R, MatLab)
- Avoid stating that data files are “available upon request”

Please indicate the figures or tables for which source data files have been provided:

The EM maps and atomic models have been deposited with the Electron Microscopy Data Bank (accession numbers EMD-20192, EMD-20194, and the Protein Data Back (entry codes 6OT2 and 6OT5) respectively.
